# Using three statistical methods to analyze the association between aldehyde exposure and markers of inflammation and oxidative stress

**DOI:** 10.1007/s11356-023-27717-4

**Published:** 2023-06-07

**Authors:** Xiaodong Zang, Wengang Qin, Yingying Xiong, Anlan Xu, Hesuyuan Huang, Tao Fang, Xiaowei Zang, Mingwu Chen

**Affiliations:** 1 Department of Pediatrics, The First Affiliated Hospital of USTC, Division of Life Sciences and Medicine, University of Science and Technology of China, Hefei, 230001 Anhui China; 2 Department of Pediatrics, Provincial Hospital Affiliated to Anhui Medical University, Hefei, 230001 Anhui China; 3 Orthopedics Department, Peking University Shougang Hospital, Beijing, 100144 China; 4 College of Safety Science and Engineering, Nanjing Tech University, Nanjing, 211816 China

**Keywords:** Aldehyde, Inflammation and oxidative stress, Multipollutant, Weighted quantile sum (WQS) regression, Bayesian kernel machine regression (BKMR)

## Abstract

**Background:**

Exposure to aldehydes has been linked to adverse health outcomes such as inflammation and oxidative stress, but research on the effects of these compounds is limited. This study is aimed at assessing the association between aldehyde exposure and markers of inflammation and oxidative stress.

**Methods:**

The study used data from the NHANES 2013–2014 survey (*n* = 766) and employed multivariate linear models to investigate the relationship between aldehyde compounds and various markers of inflammation (alkaline phosphatase (ALP) level, absolute neutrophil count (ANC), and lymphocyte count) and oxidative stress (bilirubin, albumin, and iron levels) while controlling for other relevant factors. In addition to generalized linear regression, weighted quantile sum (WQS) and Bayesian kernel machine regression (BKMR) analyses were applied to examine the single or overall effect of aldehyde compounds on the outcomes.

**Results:**

In the multivariate linear regression model, each 1 standard deviation (SD) change in propanaldehyde and butyraldehyde was significantly associated with increases in serum iron levels (beta and 95% confidence interval, 3.25 (0.24, 6.27) and 8.40 (0.97, 15.83), respectively) and the lymphocyte count (0.10 (0.04, 0.16) and 0.18 (0.03, 0.34), respectively). In the WQS regression model, a significant association was discovered between the WQS index and both the albumin and iron levels. Furthermore, the results of the BKMR analysis showed that the overall impact of aldehyde compounds was significantly and positively correlated with the lymphocyte count, as well as the levels of albumin and iron, suggesting that these compounds may contribute to increased oxidative stress.

**Conclusions:**

This study reveals the close association between single or overall aldehyde compounds and markers of chronic inflammation and oxidative stress, which has essential guiding value for exploring the impact of environmental pollutants on population health.

**Supplementary Information:**

The online version contains supplementary material available at 10.1007/s11356-023-27717-4.

## Introduction

Human exposure to aldehydes can come from industrial waste, air pollution, food additives, tobacco smoke, and internal biological processes (Ahmed Laskar &Younus 2019, O’Brien et al. 2005). These chemicals have been linked to various health problems and diseases, including cancer, birth defects, genetic mutations, diabetes, hypertension, myeloid leukemia, and neurodegenerative diseases (Tan et al., 2018, Uchida, 2000, Coggon et al., 2014, Spencer, 2018). While the precise role of aldehyde exposure in disease progression remains unclear, it is thought that their impact on inflammation and oxidative stress could be crucial factors. Animal studies have found a strong connection between aldehydes and inflammation and/or oxidative stress (Duan et al. 2020, Ong et al. 2012). For instance, mice exposed to acrolein for 4 days experienced a significant inflammatory response, marked by elevated levels of macrophages, neutrophils, and cytokines such as TNF-α, IL-1β, IL-6, MCP-1, and IFN-γ (Ong et al. 2012). Moreover, excessive alcohol consumption has been reported to result in the production and buildup of a considerable amount of acetaldehyde within the body. This accumulation induces oxidative stress, apoptosis, and inflammation in neuronal cells, ultimately leading to a decline in learning and cognitive abilities (Yan & Zhao 2020). However, the relationship between aldehyde exposure and inflammation and oxidative stress markers in humans has not yet been thoroughly studied. Additionally, previous research has typically focused on the impact of a single aldehyde or a group of aldehydes, rather than overall aldehyde exposure (Augenreich et al. 2020, Cho et al. 2017). Therefore, a more comprehensive understanding of the relationship between aldehyde exposure and inflammation and oxidative stress is needed.

Inflammation plays a crucial role in the pathogenesis of various diseases, including atherosclerosis, hypertension (Wenzel et al. 2019), and diabetes (Bharath et al. 2020). Environmental contaminants, such as inhaled pollutants (silica or asbestos) and endocrine-disrupting chemicals, can alter gene expression patterns and lead to inflammation (Dostert et al. 2008, Xu et al. 2020b). The absolute neutrophil count (ANC) is often used as a diagnostic tool to determine the presence of infection, acute inflammation, and exposure to toxic substances in the environment (Kubesch et al. 2015). Previous studies have shown a positive correlation between phthalate exposure and ANC (Ferguson et al. 2012). Furthermore, chronic inflammation is a type of prolonged inflammation that can last for weeks or even years and is caused by the persistence of the inciting factor in affected tissue (Ferrero-Miliani et al. 2007). The main responsible cells are monocytes, lymphocytes, macrophages, plasma cells, and eosinophils. Among these cells, lymphocytes coordinate the immune system’s response and play a central role in cell-mediated immunity (Bosire et al. 2013). Additionally, alkaline phosphatase (ALP) is commonly used as a marker of early stages of osteogenic differentiation, but recently, it has been utilized as a marker of inflammation in atherosclerosis and peripheral vascular disease (Webber et al. 2010). In this way, both lymphocyte count and ALP can serve as important diagnostic tools for determining the presence of chronic inflammation in various diseases. However, research on the relationship between individual or combined levels of aldehydes and ALP or ANC is limited.

Oxidative stress refers to the imbalance between the excessive accumulation of reactive oxygen species (ROS) and the ability of antioxidant defense systems to eliminate them, resulting in excessive ROS concentrations (Zhang et al. 2016).

In response to excessive ROS production, cells produce antioxidant molecules in sufficient quantities to prevent oxidative stress damage (Musaogullari & Chai 2020). Bilirubin has been identified as an antioxidant, and its elevation has been directly linked to lipid pro-oxidant activity (Gazzin et al. 2016). Previous studies have shown a significant positive correlation between exposure to environmental pollutants, such as polyfluoroalkyl substances and phthalates, and bilirubin levels (Ferguson et al. 2012, Omoike et al. 2021). In addition, iron overload increases oxidative stress and can result in mitochondrial dysfunction, reducing ATP production in cardiomyocytes (Wongjaikam et al. 2017). Albumin, on the other hand, is a negative acute-phase reactant, and its serum levels may reflect ongoing systemic inflammation (Winter et al. 2007, Yeh et al. 2018, Yin et al. 2018).

In daily life, people are inevitably exposed to a mixture of multiple pollutants. Traditional generalized linear regression analysis treats each component as an independent exposure, ignoring the possible complex interactions between mixtures.

Therefore, the primary aim of this study was to examine the association between aldehyde exposure and markers of inflammation and oxidative stress. Secondly, we employed supervised machine learning methods to pinpoint compounds of potential importance in mixtures and evaluate their influence on the observed outcomes. By identifying crucial compounds with possible significance, researchers can concentrate their efforts on these substances for further validation and investigation, potentially expediting the process of uncovering the underlying biological mechanisms.

## Methods

### Study population

**Fig. 1:**
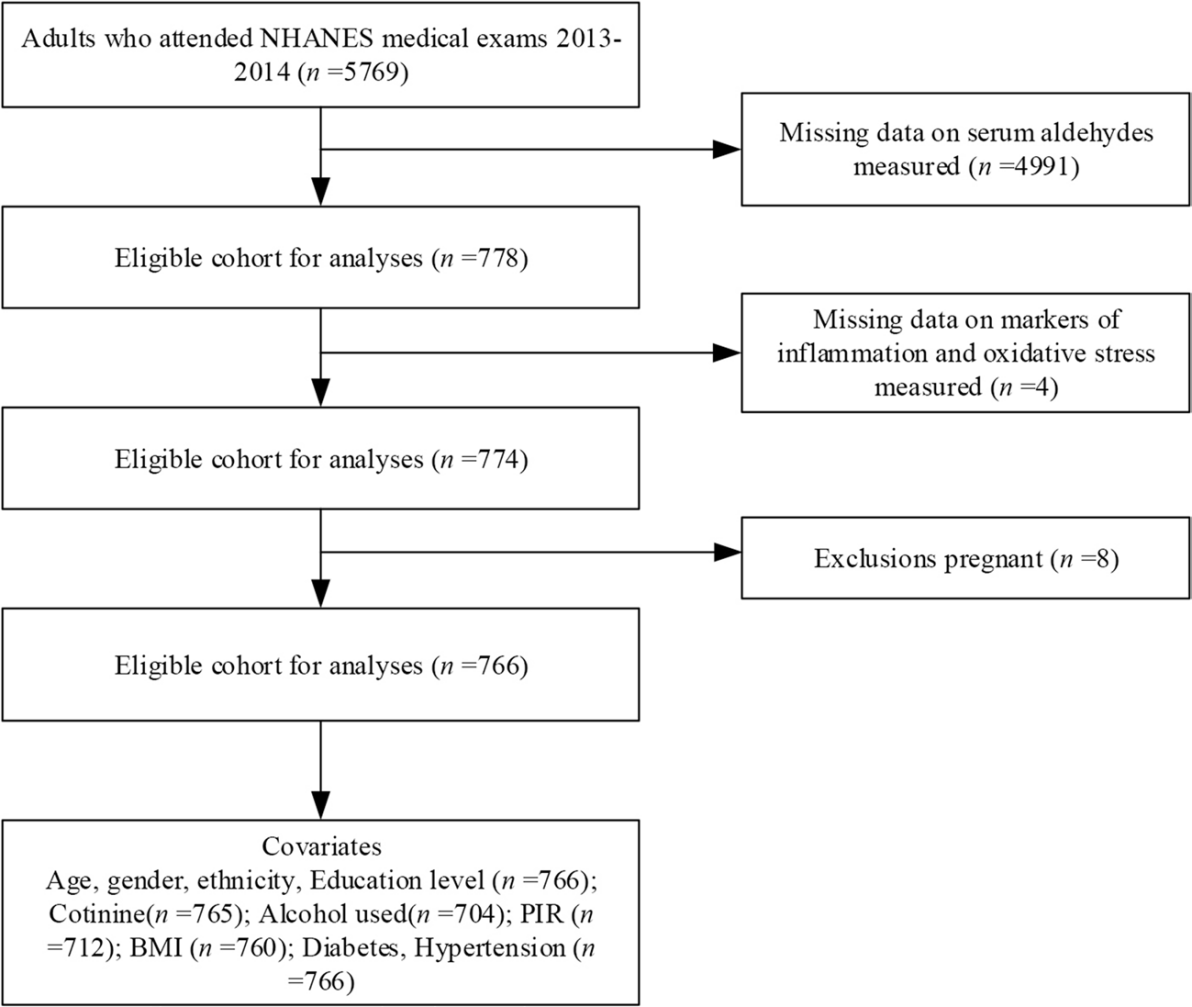
Participant characteristics (*N* = 766) from the NHANES 2013–2014

The National Center for Health Statistics (NCHS) conducts the National Health and Nutrition Examination Survey (NHANES) to assess the health, nutritional, and lifestyle status of the civilian population. (https://wwwn.cdc.gov/nchs/nhanes/Default.aspx). The NHANES survey employs a complex, stratified, and multistage sampling method that is executed every alternate year. A total of 5769 adults aged 20 or older were enrolled in the 2013–2014 NHANES study. Individuals without serum aldehyde chemical analysis data (*n* = 4991) were excluded from the study population. Furthermore, participants with any missing values for markers of inflammation (ALP, ANC, and lymphocyte count) and oxidative stress (serum bilirubin, albumin, and iron levels) were removed (*n* = 4). Pregnant individuals were also omitted from the study (*n* = 8), resulting in a final study sample size of 766 participants. The study flowchart and exclusion criteria are summarized in Fig. 1. The project was approved by the NCHS Institutional Review Board. Participants in the NHANES study were required to provide written informed consent at the time of recruitment. A detailed description of the survey and its protocols can be found on the NHANES website.

### Assessment of aldehyde levels

NHANES mobile examination centers were utilized to obtain blood specimens. Serum aldehydes were quantified through solid-phase microextraction headspace sampling in conjunction with gas chromatography and high-resolution mass spectrometry. Since aldehydes tend to react with biomolecules and generate various products, including Schiff base protein adducts, the free aldehydes released into biological samples due to Schiff base protein adducts at a low pH (~ 3) were investigated. The participants’ serum samples were analyzed for quantification of a dozen aldehydes, including crotonaldehyde, o-tolualdehyde, isopentanaldehyde, propanaldehyde, hexanaldehyde, pentanaldehyde, butyraldehyde, decanaldehyde, octanaldehyde, benzaldehyde, heptanaldehyde, and nonanaldehyde. Of the 12 measured aldehydes, we selected the 6 aldehydes that were detected in at least 85% of the samples. The detection limit and rate of these aldehydes are reported in supplementary material S1. In accordance with the guidelines of the NHANES, values of aldehydes below the limit of detection (LOD) were assigned a value equal to LOD divided by the square root of two. Further experimental information can be found on the NHANES website (https://wwwn.cdc.gov/Nchs/Nhanes/2013-2014/ALD_H.htm).

### Outcome variables

The outcome variables included markers of inflammation (levels of ALP, ANC, and lymphocyte count) and markers of oxidative stress (levels of serum bilirubin, albumin, and iron). The procedures for measurement can be found in the “Laboratory Methods” section of the official website of the NHANES (https://www.cdc.gov/nchs/nhanes/search/datapage.aspx?Component=Laboratory&Cycle=2013-2014), which provides comprehensive details on the methodology used.

### Covariates

Covariates were selected based on their availability and potential relationship with aldehydes, inflammation, and oxidative stress. Demographic information, such as age, sex, race/ethnicity (categorized as Mexican American, non-Hispanic White, non-Hispanic Black, other Hispanic, and other race), education level (categorized as less than 9th grade, 9th–11th grade, high school, college, or graduate), income (as a proportion of poverty level, PIR), body mass index (BMI), alcohol use (classified as at least 12 drinks per year or not), and diabetes mellitus (yes/no), was collected through NHANES. Hypertension was defined as a systolic blood pressure of 140 mmHg or higher and diastolic blood pressure of 90 mmHg or higher or self-reported use of anti-hypertensive medication. Serum cotinine levels were determined using standard laboratory procedures, as described in our previous studies (Zang et al. 2018, Zang et al. 2021).

### Statistical analysis

The participants’ demographic characteristics and concentrations of biomarkers were summarized using descriptive statistics. All analyses of aldehydes and serum cotinine were transformed by natural-log transformation (ln). Associations between aldehydes were evaluated using Spearman correlation.

### Statistical model 1: multivariate linear regression

Multivariate linear regression analysis was applied to analyze the associations between serum aldehyde concentrations and outcome variables. Three different models were built (crude mode: no adjustment; model I: adjusted for sex and age; model II: adjusted for sex, age, race/ethnicity, education level, BMI, PIR, alcohol use, and serum cotinine level; and model III: model II with additional adjustment for diabetes and hypertension. Multiplicative interaction was assessed using likelihood ratio tests. To account for the complex NHANES survey design, sampling weights were utilized following the NCHS recommendation. All statistical analyses were performed using R software (Version 4.0.3, R Foundation for Statistical Computing, Vienna, Austria).

### Statistical model 2: weighted quantile sum (WQS) regression

WQS is a regression model that estimates the overall impact of a mixture on a specific outcome based on a chemical-specific, empirically weighted index. The index is calculated using quantiles of chemicals and is weighted based on their relative importance in the mixture (Carrico et al. 2015). The method helps in identifying potentially toxic substances (Carrico et al. 2015, Gibson et al. 2019). To determine the weights, the training set data are divided into bootstrap samples, and a numerical optimization algorithm incorporating nonlinearity is employed to estimate the weights. The final index is calculated by averaging the weights across the bootstrap samples. The significance of the weighted quantile sum index is tested on the validation set (60% of the available data). The study set an a priori cutoff point of 0.16, which indicates possible important mixtures. However, other factors, such as variability and the degree of variance accounted for, should also be considered. To improve the comprehensibility of the index, a positive constraint was imposed on the model. The study conducted 10,000 bootstrapped iterations on the training set, resulting in optimized weights for the nonlinear model. The WQS regression was implemented using the “gWQS” package in the R statistical computing environment.

### Statistical model 3: Bayesian kernel machine regression (BKMR)

We estimated the exposure-response relationship between aldehydes and our outcomes (ALP level, ANC, lymphocyte count, and serum bilirubin, albumin, and iron levels) using the BKMR model (Bobb et al. 2015). This semiparametric approach allows for nonlinear and nonadditive relationships and provides measures of variable importance. The BKMR model enabled us to calculate the correlation between specific chemical exposures and outcomes in terms of their exposure-response relationships (Bobb et al. 2018). We utilized the Gaussian kernel function and fit the model using 50,000 iterations of the Markov chain Monte Carlo method. To determine the overall impact of the six aldehydes, we compared the estimated changes at a specific quantile to the concurrent changes in all aldehydes at the median (50th percentile). We also calculated the single-exposure effect by altering individual aldehyde levels from the 25th to 75th percentile. The same covariables were adjusted in the BKMR analysis as those used in the linear regression analysis (model III). The analysis was performed using R software (Version 4.0.3, R Foundation for Statistical Computing, Vienna, Austria) and the brms package on an Amazon EC2 instance with 16 logical processors and 32 GB of RAM.

## Results

The baseline characteristics of all 776 subjects in this study are presented in Table 1. Overall, the mean age was 49.22 ±16.94 years; 47.39% of the study participants were female, and 42.17% were non-Hispanic white. The mean values of bilirubin, albumin, iron, ALP, ANC, and lymphocyte count were 0.62 ± 0.28 mg/dL, 4.27 ± 0.31 g/dL, 83.02 ± 35.03 μg/dL, 65.53 ± 20.05 U/L, 7.39 ± 2.30 × 10^9/L, and 2.14 ± 0.72 × 10^9/L, respectively. The median serum concentrations of propanaldehyde, isopentanaldehyde, hexanaldehyde, heptanaldehyde, butyraldehyde, and benzaldehyde were 1.97 ng/mL, 0.44 ng/mL, 2.19 ng/mL, 0.51 ng/mL, 0.54 ng/mL, and 1.33 ng/mL, respectively. There were significant correlations (*P* < 0.05) among the five chemicals, except for the correlations between benzaldehyde and butyraldehyde (*p* = 0.59) and between benzaldehyde and isopentanaldehyde (*p* = 0.29). Furthermore, propanaldehyde was highly correlated with isopentanaldehyde (*r* = 0.54) (Supplementary S2).

**Table 1:** Participant characteristics (*N* = 766) from the NHANES 2013–2014

Characteristic	Overall
Age (years), mean and SD	49.22 ± 16.94
Sex, % and *n*	
Male	363 (47.39%)
Female	403 (52.61%)
Ethnicity, % and *n*	
Mexican American	144 (18.80%)
Other Hispanic	70 (9.14%)
Non-Hispanic White	323 (42.17%)
Non-Hispanic Black	120 (15.67%)
Other race	109 (14.23%)
Serum cotinine, ng/mL	0.04 (0.01–0.88)
Education, % and *n*	
Less than 9th grade	77 (10.05%)
9–11th grade	92 (12.01%)
High-school graduate	169 (22.06%)
Some college or AA degree	226 (29.50%)
College graduate or above	202 (26.37%)
BMI (kg/m^2^), % and *n*	
< 25	216 (28.42%)
25–30	258 (33.95%)
≥ 30	286 (37.63%)
Alcohol, % and *n*	
Non-drinker	180 (25.57%)
Drinker	524 (74.43%)
Diabetes, % and *n*	
No	671 (87.60%)
Yes	95 (12.40%)
Hypertension, % and *n*	
No	489 (63.84%)
Yes	277 (36.16%)
PIR, % and *n*	
≤ 1	177 (24.86%)
> 1	535 (75.14%)
Propanaldehyde, ng/mL	1.97 (1.51–2.50)
Isopentanaldehyde, ng/mL	0.44 (0.32–0.70)
Hexanaldehyde, ng/mL	2.19 (1.77–2.70)
Heptanaldehyde, ng/mL	0.51 (0.43–0.59)
Butyraldehyde, ng/mL	0.54 (0.40–0.70)
Benzaldehyde, ng/mL	1.33 (0.87–1.89)
Bilirubin, mg/dL	0.62 ± 0.28
Albumin, g/dL	4.27 ± 0.31
Iron, μg/dL	83.02 ± 35.03
Alkaline phosphatase, U/L	65.53 ± 20.05
Absolute neutrophil count, 10^9^/L	7.39 ± 2.30
Lymphocyte count, 10^9^/L	2.14 ± 0.72

Continuous variables are presented as the mean ± SD or median (interquartile range) and sample size (percentage) for categorical variables

### Multivariate linear regression

The results between aldehydes and markers of inflammation are presented in Supplementary S3 and Table 2. After controlling for confounders, there was no significant association between aldehydes and the ALP level or ANC in the fully adjusted model (Supplementary S3). In contrast, a significant association was obtained between aldehydes and the lymphocyte count. The results of linear regression analysis showing the relationship between changes in lymphocyte count and serum aldehydes are presented in Table 2. In the fully adjusted model (model III), which took into consideration variables such as age, ethnicity/race, gender, BMI, PIR, alcohol consumption, serum cotinine levels, diabetes, and hypertension, it was found that a 1-SD increase in serum propanaldehyde, isopentanaldehyde, and butyraldehyde was correlated with a 0.10 (95% CI 0.04, 0.16), 0.23 (95% CI 0.08, 0.37), and 0.18 (95% CI 0.03, 0.34) increase in lymphocyte count, respectively.

**Table 2:** Association between serum levels of aldehydes and the lymphocyte count in the multivariate linear regression model

Exposure	Non-adjusted	*P*	Model 1	*P*	Model 2	*P*	Model 3	*P*
	OR (95% CI)		OR (95% CI)		OR (95% CI)		OR (95% CI)	
Lymphocyte count								
Ln (propanaldehyde, ng/mL)	0.12 (0.06, 0.17)	< 0.001	0.11 (0.06, 0.16)	< 0.001	0.10 (0.04, 0.16)	< 0.01	**0.10 (0.04, 0.16)**	**< 0.01**
Propanaldehyde (quartiles)								
Q1	1 (reference)		1 (reference)		1 (reference)		1 (reference)	
Q2	− 0.03 (− 0.17, 0.12)	0.73	− 0.02 (− 0.16, 0.13)	0.81	− 0.05 (− 0.21, 0.10)	0.50	− 0.05 (− 0.21, 0.10)	0.5
Q3	0.04 (− 0.11, 0.18)	0.61	0.05 (− 0.09, 0.19)	0.47	0.00 (− 0.15, 0.16)	0.96	− 0.01 (− 0.16, 0.15)	0.92
Q4	0.31 (0.16, 0.45)	< 0.001	0.29 (0.16, 0.43)	< 0.001	0.23 (0.07, 0.39)	< 0.01	0.23 (0.08, 0.39)	< 0.01
*P* for trend		< 0.001		< 0.001		< 0.01		< 0.01
Ln (isopentanaldehyde, ng/mL)	0.22 (0.12, 0.32)	< 0.001	0.20 (0.10, 0.29)	< 0.001	0.23 (0.09, 0.37)	< 0.01	**0.23 (0.08, 0.37)**	**< 0.01**
Isopentanaldehyde (quartiles)								
Q1	1 (reference)		1 (reference)		1 (reference)		1 (reference)	
Q2	− 0.15 (− 0.30, 0.01)	0.06	− 0.10 (− 0.25, 0.05)	0.19	− 0.12 (− 0.28, 0.05)	0.16	− 0.13 (− 0.29, 0.03)	0.12
Q3	− 0.03 (− 0.18, 0.12)	0.71	0.03 (− 0.12, 0.18)	0.68	− 0.01 (− 0.17, 0.15)	0.93	− 0.01 (− 0.17, 0.15)	0.92
Q4	0.13 (− 0.01, 0.28)	0.07	0.17 (0.03, 0.31)	0.02	0.08 (− 0.11, 0.27)	0.43	0.06 (− 0.13, 0.26)	0.52
*P* for trend		0.01		< 0.01		0.31		0.35
Ln (hexanaldehyde, ng/mL)	0.02 (− 0.00, 0.04)	0.06	0.02 (− 0.00, 0.04)	0.08	0.02 (− 0.00, 0.04)	0.12	0.02 (− 0.00, 0.04)	0.11
Hexanaldehyde (quartiles)								
Q1	1 (reference)		1 (reference)		1 (reference)		1 (reference)	
Q2	0.01 (− 0.14, 0.16)	0.87	0.01 (− 0.13, 0.16)	0.85	0.00 (− 0.16, 0.16)	0.99	− 0.01 (− 0.17, 0.15)	0.89
Q3	0.06 (− 0.09, 0.21)	0.42	0.05 (− 0.09, 0.20)	0.48	0.09 (− 0.07, 0.24)	0.27	0.08 (− 0.08, 0.23)	0.34
Q4	0.16 (0.02, 0.31)	0.03	0.15 (0.01, 0.29)	0.04	0.14 (− 0.01, 0.30)	0.07	0.13 (− 0.03, 0.28)	0.11
*P* for trend		0.02		0.03		0.04		0.06
Ln (heptanaldehyde, ng/mL)	0.26 (− 0.01, 0.53)	0.06	0.17 (− 0.09, 0.44)	0.20	0.15 (− 0.14, 0.43)	0.31	0.16 (− 0.13, 0.44)	0.28
Heptanaldehyde (quartiles)								
Q1	1 (reference)		1 (reference)		1 (reference)		1 (reference)	
Q2	0.06 (− 0.09, 0.21)	0.44	0.06 (− 0.09, 0.20)	0.45	0.06 (− 0.09, 0.22)	0.43	0.06 (− 0.10, 0.22)	0.45
Q3	0.03 (− 0.12, 0.17)	0.74	− 0.00 (− 0.15, 0.14)	0.96	0.02 (− 0.14, 0.18)	0.83	0.02 (− 0.14, 0.18)	0.81
Q4	0.12 (− 0.03, 0.27)	0.13	0.08 (− 0.07, 0.23)	0.28	0.07 (− 0.10, 0.23)	0.41	0.07 (− 0.10, 0.23)	0.42
*P* for trend		0.2		0.46		0.58		0.57
Ln (butyraldehyde, ng/mL)	0.15 (− 0.02, 0.31)	0.09	0.20 (0.05, 0.35)	0.01	0.16 (− 0.01, 0.32)	0.07	**0.18 (0.03, 0.34)**	**0.02**
Butyraldehyde (quartiles)								
Q1	1 (reference)		1 (reference)		1 (reference)		1 (reference)	
Q2	0.13 (− 0.03, 0.29)	0.11	0.13 (− 0.02, 0.29)	0.09	0.07 (− 0.09, 0.23)	0.40	0.07 (− 0.09, 0.23)	0.39
Q3	0.12 (− 0.03, 0.27)	0.12	0.14 (− 0.01, 0.29)	0.07	0.14 (− 0.02, 0.30)	0.09	0.14 (− 0.02, 0.29)	0.09
Q4	0.25 (0.10, 0.40)	< 0.01	0.27 (0.12, 0.42)	< 0.01	0.24 (0.07, 0.40)	< 0.01	0.22 (0.06, 0.38)	0.01
*P* for trend		< 0.01		< 0.01		< 0.01		< 0.01
Ln (benzaldehyde, ng/mL)	− 0.00 (− 0.03, 0.03)	0.88	− 0.01 (− 0.03, 0.02)	0.64	− 0.01 (− 0.03, 0.02)	0.71	− 0.01 (− 0.04, 0.02)	0.65
Benzaldehyde (quartiles)								
Q1	1 (reference)		1 (reference)		1 (reference)		1 (reference)	
Q2	0.07 (− 0.07, 0.21)	0.34	0.05 (− 0.09, 0.18)	0.50	0.10 (− 0.05, 0.25)	0.19	0.10 (− 0.05, 0.24)	0.20
Q3	0.03 (− 0.11, 0.17)	0.68	0.00 (− 0.14, 0.14)	0.98	0.05 (− 0.10, 0.20)	0.53	0.05 (− 0.10, 0.20)	0.50
Q4	0.05 (− 0.10, 0.20)	0.51	0.03 (− 0.12, 0.17)	0.71	0.05 (− 0.11, 0.21)	0.52	0.04 (− 0.11, 0.20)	0.58
*P* for trend		0.61		0.86		0.6		0.64

Model 1: adjusted for age and sex. Model 2: model 1 plus race/ethnicity, family PIR, education level, serum cotinine level, BMI category, and past-year alcohol consumption. Model 3: model 2 plus diabetes and hypertension. Bold values indicate statistical significance (*P* < 0.05). *PIR* poverty income ratio, *OR* odds ratio, *CI* confidence interval

In addition, a significant interaction between isopentanaldehyde and sex was observed for the lymphocyte count (*P* for interaction = 0.03) (Supplementary S5). After grouping the serum aldehyde levels into quintiles, the individuals with the highest quintile levels of propanaldehyde or butyraldehyde exhibited a 0.23 (95% CI 0.08, 0.39) and 0.22 (95% CI: 0.06, 0.38) increase in lymphocyte count, respectively, compared to those with the corresponding lowest quintile. A significant trend was observed in the *P* values for the propanaldehyde and butyraldehyde models from the lowest to the highest quintile.

The results between aldehydes and markers of oxidative stress are presented in Supplementary S4 and Table 3. No significant association was found between aldehydes and bilirubin in the fully adjusted model (Supplementary S4). Similarly, we did not find a correlation between aldehydes and albumin after controlling for confounders. However, we found a significant association of aldehydes with serum iron. Overall, in adjusted model III, propanaldehyde, butyraldehyde, and benzaldehyde were associated with a significant increase in serum iron concentrations (Table 3). After adjusting for the covariates, a 1-SD change in propanaldehyde was associated with a statistically significant increase in serum iron levels, with *β* ranging from 0.24 to 6.27. Similarly, a 1-SD change in butyraldehyde was also associated with a significant increase in serum iron levels, with *β* ranging from 0.97 to 15.83. Furthermore, a 1-SD change in benzaldehyde was found to significantly raise serum iron levels, with *β* ranging from 0.00 to 2.78.

**Table 3:** Association between serum levels of aldehydes and serum iron levels in the multivariate linear regression model

Exposure	Non-adjusted	*P*	Model 1	*P*	Model 2	*P*	Model 3	*P*
	OR (95% CI)		OR (95% CI)		OR (95% CI)		OR (95% CI)	
Iron								
Ln (propanaldehyde, ng/mL)	4.43 (1.73, 7.13)	< 0.01	3.92 (1.24, 6.59)	< 0.01	3.27 (0.26, 6.28)	0.03	**3.25 (0.24, 6.27)**	**0.03**
Propanaldehyde (quartiles)								
Q1	1 (reference)		1 (reference)		1 (reference)		1 (reference)	
Q2	7.04 (− 0.11, 14.19)	0.05	6.00 (− 1.07, 13.06)	0.1	5.37 (− 2.12, 12.87)	0.16	5.32 (− 2.18, 12.82	0.16
Q3	11.49 (4.44, 18.54)	< 0.01	10.23 (3.26, 17.20)	< 0.01	10.87 (3.40, 18.34)	< 0.01	10.85 (3.36, 18.34	0
Q4	12.33 (5.42, 19.24)	< 0.01	10.92 (4.08, 17.77)	< 0.01	9.86 (2.22, 17.50)	0.01	9.88 (2.23, 17.53	0.01
*P* for trend		< 0.01		< 0.01		< 0.01		< 0.01
Ln (isopentanaldehyde, ng/mL)	2.94 (− 1.85, 7.74)	0.23	1.84 (− 2.92, 6.60	0.45	− 0.48 (− 7.40, 6.44)	0.89	− 0.52 (− 7.44, 6.41	0.88
Isopentanaldehyde (quartiles)								
Q1	1 (reference)		1 (reference)		1 (reference)		1 (reference)	
Q2	7.29 (− 0.08, 14.66	0.05	6.47 (− 0.82, 13.77	0.08	5.71 (− 2.13, 13.55)	0.15	5.72 (− 2.14, 13.58)	0.15
Q3	6.49 (− 0.78, 13.77	0.08	4.90 (− 2.34, 12.14	0.18	4.26 (− 3.54, 12.06)	0.28	4.04 (− 3.77, 11.86)	0.31
Q4	6.87 (− 0.15, 13.88	0.06	4.82 (− 2.15, 11.79	0.18	2.82 (− 6.48, 12.13)	0.55	2.92 (− 6.40, 12.24)	0.54
*P* for trend		0.1		0.31		0.55		0.56
Ln (hexanaldehyde, ng/mL)	− 0.08 (− 1.11, 0.94	0.87	0.01 (− 0.99, 1.02	0.98	0.07 (− 0.94, 1.07	0.9	0.03 (− 0.99, 1.04)	0.96
Hexanaldehyde (quartiles)								
Q1	1 (reference)		1 (reference)		1 (reference)		1 (reference)	
Q2	8.69 (1.47, 15.92)	0.02	8.18 (1.06, 15.29)	0.02	7.59 (− 0.06, 15.23)	0.05	7.65 (− 0.01, 15.32)	0.05
Q3	9.57 (2.46, 16.69)	0.01	9.75 (2.74, 16.75)	0.01	8.51 (1.02, 16.00)	0.03	8.62 (1.12, 16.13)	0.02
Q4	9.89 (2.93, 16.84)	0.01	9.40 (2.55, 16.26)	0.01	10.08 (2.62, 17.53)	0.01	10.28 (2.79, 17.77)	0.01
*P* for trend		0.01		0.01		0.01		0.01
Ln (heptanaldehyde, ng/mL)	5.02 (− 8.06, 18.10)	0.45	3.49 (− 9.47, 16.45)	0.6	− 2.01 (− 15.70, 11.67)	0.77	− 2.11 (− 15.81, 11.58)	0.76
Heptanaldehyde (quartiles)								
Q1	1 (reference)		1 (reference)		1 (reference)		1 (reference)	
Q2	2.50 (− 4.69, 9.69)	0.50	2.85 (− 4.24, 9.94)	0.43	1.21 (− 6.37, 8.79)	0.75	1.00 (− 6.60, 8.59)	0.80
Q3	3.36 (− 3.83, 10.55)	0.36	3.40 (− 3.70, 10.50)	0.35	1.28 (− 6.35, 8.92)	0.74	1.30 (− 6.33, 8.94)	0.74
Q4	6.83 (− 0.57, 14.23)	0.07	4.59 (− 2.79, 11.97)	0.22	1.21 (− 6.72, 9.14)	0.77	1.32 (− 6.62, 9.25)	0.74
*P* for trend		0.07		0.23		0.78		0.74
Ln (butyraldehyde, ng/mL)	8.03 (− 0.05, 16.10)	0.05	6.49 (− 0.87, 13.85)	0.08	7.93 (− 0.14, 16.00)	0.05	**8.40 (0.97, 15.83)**	**0.03**
Butyraldehyde (quartiles)								
Q1	1 (reference)		1 (reference)		1 (reference)		1 (reference)	
Q2	− 0.28 (− 7.89, 7.33)	0.94	− 1.00 (− 8.51, 6.50)	0.79	0.34 (− 7.55, 8.23)	0.93	0.50 (− 7.40, 8.40)	0.90
Q3	7.51 (0.19, 14.83)	0.04	6.20 (− 1.03, 13.44)	0.09	5.68 (− 1.97, 13.32)	0.15	5.76 (− 1.89, 13.40)	0.14
Q4	7.38 (0.03, 14.74)	0.05	5.37 (− 1.93, 12.67)	0.15	4.19 (− 3.63, 12.00)	0.29	4.29 (− 3.55, 12.13)	0.28
*P* for trend		0.01		0.04		0.15		0.15
Ln (benzaldehyde, ng/mL)	1.40 (0.02, 2.78)	0.05	1.72 (0.36, 3.09)	0.01	1.39 (− 0.00, 2.78)	0.05	**1.39 (0.00, 2.78)**	**< 0.05**
Benzaldehyde (quartiles)								
Q1	1 (reference)		1 (reference)		1 (reference)		1 (reference)	
Q2	3.97 (− 2.69, 10.62)	0.24	4.35 (− 2.19, 10.89)	0.19	1.74 (− 5.35, 8.84)	0.63	1.80 (− 5.30, 8.90)	0.62
Q3	7.10 (0.28, 13.91)	0.04	8.18 (1.48, 14.89)	0.02	5.79 (− 1.46, 13.04)	0.12	5.66 (− 1.59, 12.92)	0.13
Q4	12.72 (5.63, 19.82)	< 0.01	14.38 (7.38, 21.37)	< 0.0001	12.69 (5.10, 20.28)	< 0.001	12.88 (5.28, 20.48)	< 0.001
*P* for trend		< 0.01		< 0.0001		< 0.001		< 0.001

Model 1: adjusted for age and sex. Model 2: model 1 plus race/ethnicity, family PIR, education level, serum cotinine level, BMI category, and past-year alcohol consumption. Model 3: model 2 plus diabetes and hypertension. Bold values indicate statistical significance (*P* < 0.05). *PIR* poverty income ratio, *OR* odds ratio, *CI* confidence interval

### Weighted quantile sum regression

**Fig. 2:**
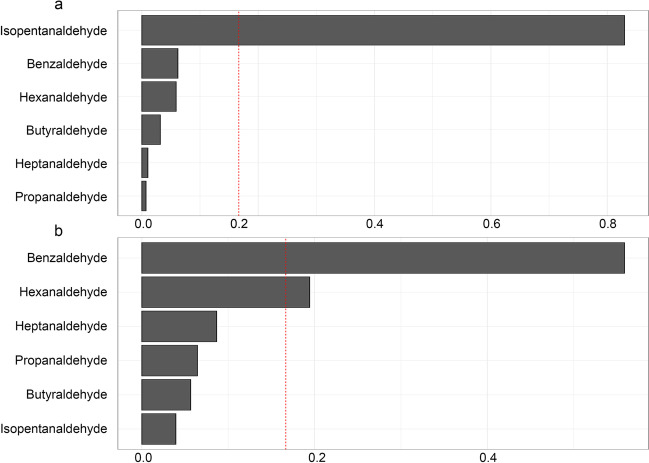
WQS model regression index weights for albumin (**a**) and iron (**b**). The bar plot shows the weights assigned to each chemical. Model adjusted for age, sex, race/ethnicity, PIR, education level, serum cotinine level, BMI, alcohol use, diabetes, and hypertension. The dashed line at 0.16 indicates the cutoff point for identifying potentially toxic agents

The mixture index coefficient in the WQS model demonstrated a positive correlation with albumin (0.02, 95% CI = 0.00 to 0.03), as shown in Fig. 2a. Only isopentanaldehyde had a weight greater than 0.16 (weight = 0.82) (Fig. 2a). Furthermore, the mixture index coefficient in the WQS model exhibited a significant positive correlation with serum iron (0.02, 95% CI = 0.00 to 0.03), as shown in Fig. 2b. Two aldehydes, benzaldehyde and hexanaldehyde, had weights greater than 0.16 (weight of benzaldehyde = 0.55 and weight of hexanaldehyde = 0.19). However, there was no correlation between the mixture index coefficient in the WQS model and the levels of ALP, ANC, lymphocyte count, or bilirubin (Supplementary S6).

### Bayesian kernel machine regression

**Fig. 3:**
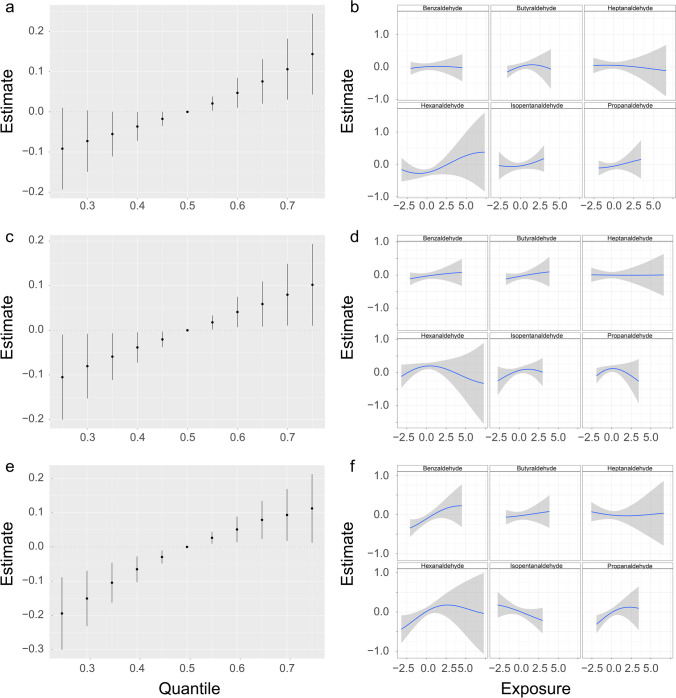
Joint effect of the aldehyde mixture on outcomes by using a BKMR model. The models were controlled for various factors, including age, sex, ethnicity, poverty index ratio, education, serum cotinine levels, body mass index, alcohol consumption, diabetes, and hypertension. **a** Overall risk (95% CI) of the mixture on the lymphocyte count when comparing all the exposures at different percentiles with their median level. **b** Association and 95% confidence intervals for each chemical exposure with the lymphocyte count while fixing other chemical exposures at their median level. **c** Overall risk (95% CI) of the mixture on albumin when comparing all the exposures at different percentiles with their median level. **d** Association and 95% confidence intervals for each chemical exposure with albumin while fixing other chemical exposures at their median level. **e** Overall risk (95% CI) of the mixture on iron when comparing all the exposures at different percentiles with their median level. **f** Association and 95% credible intervals for each chemical exposure with iron while fixing other chemical exposures at their median level

BKMR analysis was performed using a new hybrid methodology to evaluate the combined and single-exposure effects of aldehyde mixture exposure on markers of inflammation and oxidative stress (Fig. 3 and Supplementary S7). The results revealed that there was a significant increase in lymphocyte count when all aldehydes were above the 55th percentile compared to their median value, indicating a positive correlation between aldehyde exposure and lymphocyte count (Fig. 3a). Figure 3b depicts the positive associations of isopentanaldehyde, propanaldehyde, and hexanaldehyde with lymphocyte count in the BKMR models, with all other chemical exposures fixed at their median levels. As depicted in Fig. 3c, the relationship between aldehydes and albumin is shown to be positive, with albumin increasing significantly when all aldehydes were above their 55th percentile compared to when they were at their 50th percentile. Furthermore, as shown in Fig. 3d, individual exposures to isopentanaldehyde, butyraldehyde, and benzaldehyde were positively correlated with albumin when the levels of other aldehydes were held constant at their median. In addition, Fig. 3e illustrates the positive associations of aldehydes with iron in the BKMR models while controlling all other chemical exposures at their median level. This combined effect showed an increase as the level of aldehyde exposure increased. Figure 3f reveals the linearity of some independent chemical associations, with benzaldehyde being statistically significantly associated with iron and suggestive evidence of positive associations with hexanaldehyde and propanaldehyde.

## Discussion

The current study analyzed the relationship between serum aldehydes and markers of inflammation and oxidative stress in American adults using traditional linear regression analysis and two supervised machine learning approaches: WQS and BKMR models. The results showed a significant connection between individual or combined aldehyde compounds and markers of inflammation and oxidative stress.

Previous reports have shown that elevated ALP levels are associated with inflammatory diseases (Brichacek & Brown 2019, Haarhaus et al. 2017) (e.g., autoimmune diseases, lung diseases, intestinal diseases, metabolic disorders, and heart diseases). However, the cause of the increase in ALP levels is unclear, but exposure to environmental pollutants is considered a possible cause. Liu et al. suggested a significant link between fine particulate matter (PM2.5) and elevated ALP levels (Liu et al. 2017). However, our results found no correlation between a single aldehyde or overall aldehyde and the ALP level, nor was a correlation found in the WQS and BKMR models. A possible explanation is that, similar to C-reactive protein (CRP), ALP is more suitable as a marker of acute rather than chronic or persistent inflammation. A larger clinical trial is necessary to determine the generalizability of the current findings. Similar observations were found for another acute inflammatory marker, the ANC. Our results suggest that per and aldehydes are not associated with an increase in the ANC. This seems to be inconsistent with previous studies, which explored the impact of inhaling diesel exhaust, air pollution, and nanoparticles on human oxidative stress and revealed that these pollutants can significantly increase the levels of inflammatory cells (Frampton et al. 2006, Jacobs et al. 2010, Kubesch et al. 2015). A possible explanation is that the effect of aldehydes on the human body is a persistent or chronic effect rather than an acute effect.

Increased lymphocyte reactivity is observed in chronic diseases, such as diabetes, hypertension, hepatitis, and cardiovascular diseases, potentially related to repeated chronic inflammation stimulation (Jung et al. 2019, Wang et al. 2020, Li et al. 2018). Moreover, animal studies have demonstrated that lymphocyte vitality and proliferation are affected following aldehyde exposure (Poirier et al. 2002). Multivariate linear regression showed a positive association between propionaldehyde, isopentanaldehyde, and butyraldehyde and the lymphocyte count. This finding is similar to that of a previous study (Xu et al. 2020a). Although the exact processes by which aldehydes impact lymphocyte counts remain unclear, research has suggested various potential pathways. One such mechanism proposes that aldehydes may enhance oxidative stress and inflammation, subsequently causing an increase in lymphocyte count as the immune system responds to the damage induced by aldehyde exposure (Ge et al. 2020). Moreover, aldehydes might directly or indirectly influence the generation and secretion of cytokines, further contributing to the activation of the immune system (Wei et al. 2014). Xu et al. proved that serum isopentanaldehyde was significantly related to an increase in lymphocytes (Xu et al. 2020a). Here, we have added evidence that there is a significant interaction between isopentanaldehyde and sex and lymphocyte count. This correlation is even more pronounced among men. The specific mechanisms involved are unknown but may be related to the biological response to oxidative stress at varying levels of sex hormones. Considering that estrogen is a powerful antioxidant, it can reduce the body’s oxidative stress level (Vina et al. 2013). In the WQS model, although no significant correlation was observed between the combined exposure to chemicals and lymphocyte count, a rising trend was noted. In the BKMR model, the lymphocyte count was seen to rise significantly when all aldehydes were at or above their 55th percentile compared to when they were at the median value, signifying a positive relationship between exposure to aldehydes and lymphocyte count. Isopentanaldehyde, propanaldehyde, and hexanaldehyde showed a positive single-exposure effect on the lymphocyte count. The positive correlation between aldehydes and the lymphocyte count provides a basis for further research on aldehydes that may cause liver damage, especially the simultaneous increase in serum iron, one of the early indicators of liver damage.

A substantial amount of clinical data suggests that elevated iron stores can negatively impact an individual’s susceptibility to disease and response to infection and inflammation (Fourati et al. 2018). Currently, there is a scarcity of research and literature exploring the relationship between aldehydes and serum iron levels. Aldehydes, a category of organic compounds characterized by the presence of carbonyl groups, exhibit potent reducing properties that may affect proteins. Proteins involved in regulating iron levels, such as ferritin and transferrin, are composed of numerous amino acid residues. As a result, their structure and function could potentially be impacted by the presence of aldehydes. The multiple regression model found a significant association between aldehydes (benzaldehyde, propanaldehyde, and butyraldehyde) and iron. In addition, in the BKMR models, benzaldehyde was statistically significantly associated with iron, meaning benzaldehyde may be the most important compound that causes aldehydes to increase iron. To date, there is no literature on the association between benzaldehyde and serum iron.

Oxidation-related damage causes alterations to proteins and is a contributing factor in many illnesses. Serum albumin, the most prevalent protein in plasma, plays a crucial role in protecting against oxidative damage through its antioxidant properties (Anraku et al. 2013, Rabbani & Ahn 2019). The antioxidant capacity of albumin is mainly dependent on the presence of Cys-34 and its role in maintaining intravascular stability, including protection of the vascular endothelium in diseases caused by oxidative stress (Leboffe et al. 2020, Roche et al. 2008). As a result, albumin is considered a negative reactant, exhibiting extracellular antioxidant qualities due to prolonged exposure to environmental pollutants, which results in a compensatory increase. The results of this study showed that no single aldehyde compound had a statistical association with albumin in multivariate linear regression analysis. It is important to note that the generalized linear model was unable to analyze interactions between exposures, which could account for the conflicting results stemming from overlap or interactions between exposures. The BKMR model, in comparison to traditional multiple regression, is better at handling the nonlinear relationship between mixtures and their interactions. The WQS and BKMR models both showed a positive correlation between aldehyde compounds and serum albumin levels. Furthermore, isopentanaldehyde had the highest weight in both the WQS and BKMR models, suggesting that it may be a crucial compound responsible for increasing albumin levels. This finding has not yet been reported in the literature. Like serum albumin, bilirubin also acts as a powerful antioxidant by scavenging harmful free radicals and protecting the body from oxidative stress. Previous research has reported that exposure to aldehydes can increase serum bilirubin levels, but our results did not find any correlation between aldehyde compounds and bilirubin. This discrepancy may be because bilirubin has strong antioxidant properties, which can be impacted by acute and short-term exposure to aldehydes, whereas long-term and low-dose exposure may not result in a significant increase in bilirubin due to the body’s compensatory mechanisms against oxidation.

The inflammatory and oxidative stress responses elicited by aldehydes may encompass diverse pathological mechanisms (Moretto et al. 2012, Zirak et al. 2019), including (1) compromising cellular membrane integrity, leading to lipid oxidation and phosphatidylcholine synthesis, consequently provoking inflammation; (2) fostering oxidative stress through enhanced intracellular free radical production and reduced antioxidant levels, resulting in oxidative damage to intracellular proteins, nucleic acids, and lipids, followed by the induction of inflammation; (3) interacting with DNA, thereby inflicting damage and mutations that further incite inflammation; and (4) associating with proteins, inducing protein modification and oxidation that subsequently trigger inflammation. These interconnected biological mechanisms may contribute to the pathogenesis of chronic diseases, such as cancer, diabetes, cardiovascular diseases, and autoimmune disorders. For instance, aldehyde metabolites are associated with rheumatoid arthritis (RA), a chronic inflammatory disease primarily characterized by joint inflammation and degeneration. Although the etiology of RA is not yet fully understood, research suggests that it may be linked to air pollution from sources such as agriculture, fossil fuel combustion, chemical industries, and solvent use (Radu & Bungau 2021). These air pollutants contain a significant amount of aldehyde compounds, which can interact with biomacromolecules (e.g., proteins and lipids) to form highly immunogenic adducts. This process may induce the immune system to erroneously attack self-tissues, ultimately leading to the development of RA. Currently, the development of novel antioxidant therapies targeting specific pathways is a promising direction for future research (Rotariu et al. 2022). Our findings suggest that individual aldehyde compounds exert differential roles in the oxidative stress process. Therefore, future antioxidant therapies can be tailored by monitoring specific aldehyde metabolite levels, enabling the delivery of more accurate and efficacious treatment strategies for patients and ultimately enhancing the prevention and treatment outcomes of oxidative stress-related diseases.

Our study has the following advantages. Firstly, based on large-scale population data, we conducted a comprehensive investigation of the effects of individual and combined aldehyde compounds on various inflammation and oxidative stress biomarkers. By analyzing the roles of different aldehydes on distinct oxidative stress markers, we provide crucial insights for further studies on the relationship between aldehydes and specific diseases. Secondly, we employed supervised machine learning techniques to help identify compounds of potential significance in mixtures and assess the overall impact of these mixtures on the outcomes. These findings aid in identifying key substances within a multitude of mixtures, offering direction for subsequent validation studies. For instance, our research discovered that benzaldehyde might be the most crucial compound responsible for the increased iron content in aldehydes. This discovery lays a solid foundation for further investigation into aldehyde-induced liver damage, as abnormalities in iron metabolism are considered one of the early indicators of liver injury.

Our research results have the following limitations. Firstly, the cross-sectional design employed precludes the establishment of causal relationships between aldehyde exposure and inflammation and oxidative stress markers. Consequently, future investigations should incorporate longitudinal designs, as well as animal experiments, to validate the present findings and elucidate the underlying mechanisms. Secondly, our study population comprises American adults, thus limiting the direct generalizability of the results to other demographic groups. It is vital to extend the scope of investigation to encompass diverse populations from varying regions, environmental exposures, and lifestyles, in order to ascertain the global applicability of our findings and to deepen our comprehension of the worldwide impact of aldehyde exposure on human health. Thirdly, the aldehyde compound concentrations sourced from the database represent a single measurement, necessitating consideration of the inherent variability when drawing clinical inferences. Fourthly, although alternative inflammation and oxidative stress indicators, such as cytokines, CRP, and ferritin, may be informative, the absence of such data within the database precluded their incorporation into our study. Lastly, this research primarily focused on the associations between aldehyde exposure and inflammation and oxidative stress markers, without examining potential health outcomes. Subsequent studies should investigate the long-term health consequences of aldehyde exposure, including its role in the development of chronic diseases like cancer, diabetes, and cardiovascular diseases, to further explicate the clinical implications of our findings.

## Conclusion

The present study investigated the impact of exposure to a single or a combination of aldehydes on markers of inflammation and oxidative stress, suggesting a potential association between aldehyde exposure and increased chronic inflammation and oxidative stress. Furthermore, the study also established the exposure-response relationships between each type of aldehyde and markers of inflammation or oxidative stress, thereby identifying key contaminants. The results suggest that aldehydes may have a negative impact on human health, but further research is needed to better understand the mechanisms involved.

## Supplementary information

ESM 1 (DOCX 306 kb)

## Data Availability

CDC data are publicly available at https://wwwn.cdc.gov/nchs/nhanes/.
