# Supplementary material for: Using three statistical methods to analyze the association between aldehyde exposure and markers of inflammation and oxidative stress
Source: Environ Sci Pollut Res Int. 2023 Jun 7;30(32):79437–50. doi: 10.1007/s11356-023-27717-4 (PMC10313561; doi:10.1007/s11356-023-27717-4)

Supplementary S1: The detection rate.

| **Chemical** | **Sample size (n)** | | **Detection rate (%)** |
| --- | --- | --- | --- |
|  | At or above the detection limit (N) | Below lower detection limit (N) |  |
| Benzaldehyde  Crotonaldehyde  Isopentanaldehyde  o-Tolualdehyde  Propanaldehyde  Butyraldehyde  Pentanaldehyde  Hexanaldehyde  Heptanaldehyde  Octanaldehyde  Nonanaldehyde  Decanaldehyde | 1391  582  1783  63  1513  1372  592  1532  1407  226  572  11 | 77  1151  15  1498  201  368  1168  39  120  1230  962  1400 | 94.75  33.58  99.16  4.03  88.27  78.85  33.63  97.51  92.14  15.52  37.28  0.77 |

Supplementary S2: Correlation heatmap of all exposures calculated using data from 766 samples.


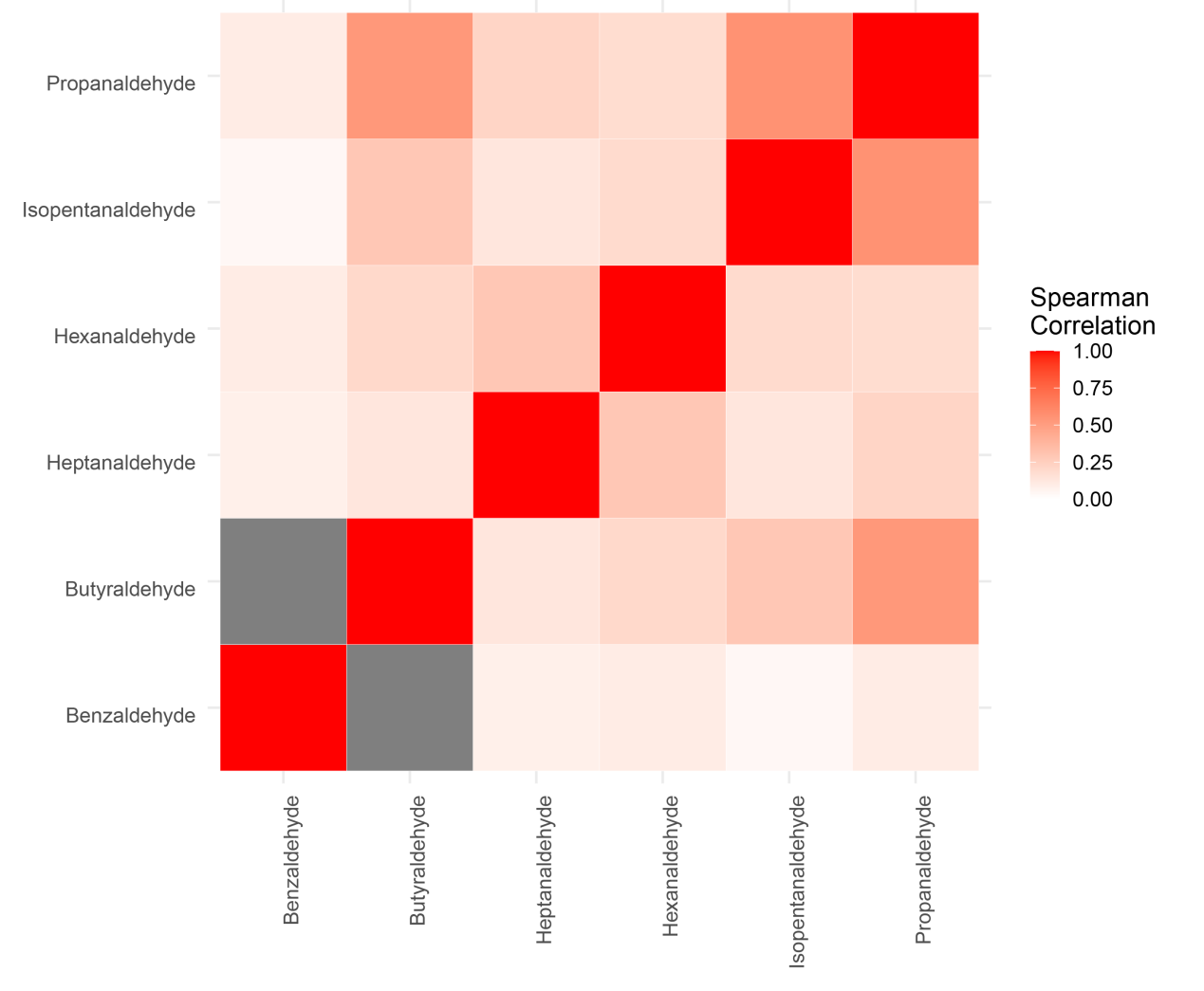


Supplementary S3: Association between serum levels of aldehydes and markers of inflammation in a multivariate linear regression model.

| **Exposure** | **Non-adjusted** | ***P*** | **Model 1** | ***P*** | **Model 2** | ***P*** | **Model 3** | ***P*** |
| --- | --- | --- | --- | --- | --- | --- | --- | --- |
|  | OR (95% CI) |  | OR (95% CI) |  | OR (95% CI) |  | OR (95% CI) |  |
| **Alkaline phosphatase** |  |  |  |  |  |  |  |  |
| Ln (Propanaldehyde, ng/mL) | 1.37 (-0.18, 2.93) | 0.08 | 1.47 (-0.09, 3.03) | 0.07 | 0.01 (-1.71, 1.73) | 0.99 | 0.02 (-1.70, 1.74) | 0.99 |
| Propanaldehyde (quartiles) |  |  |  |  |  |  |  |  |
| Q1 | 1 (reference) |  | 1 (reference) |  | 1 (reference) |  | 1 (reference) |  |
| Q2 | 1.13 (-3.00, 5.25) | 0.59 | 1.24 (-2.89, 5.38) | 0.56 | 0.27 (-4.02, 4.57) | 0.9 | 0.24 (-4.06, 4.53) | 0.91 |
| Q3 | 2.58 (-1.48, 6.65) | 0.21 | 2.70 (-1.37, 6.78) | 0.19 | 0.16 (-4.12, 4.45) | 0.94 | 0.05 (-4.24, 4.34) | 0.98 |
| Q4 | 3.03 (-0.96, 7.01) | 0.14 | 3.29 (-0.71, 7.30) | 0.11 | -0.02 (-4.40, 4.36) | 0.99 | 0.03 (-4.35, 4.42) | 0.99 |
| *P* for trend |  | 0.10 |  | 0.08 |  | 0.98 |  | 0.99 |
| Ln (Isopentanaldehyde, ng/mL) | 3.13 (0.39, 5.87) | 0.03 | 3.42 (0.66, 6.18) | 0.02 | 0.22 (-3.72, 4.16) | 0.91 | 0.17 (-3.76, 4.11) | 0.93 |
| Isopentanaldehyde (quartiles) |  |  |  |  |  |  |  |  |
| Q1 | 1 (reference) |  | 1 (reference) |  | 1 (reference) |  | 1 (reference) |  |
| Q2 | 0.77 (-3.45, 4.99) | 0.72 | 0.69 (-3.55, 4.93) | 0.75 | -1.59 (-6.06, 2.87) | 0.48 | -1.68 (-6.16, 2.79) | 0.46 |
| Q3 | 0.10 (-4.07, 4.27) | 0.96 | 0.09 (-4.11, 4.29) | 0.97 | -0.02 (-4.46, 4.42) | 0.99 | -0.23 (-4.68, 4.22) | 0.92 |
| Q4 | 3.74 (-0.28, 7.76) | 0.07 | 3.89 (-0.15, 7.94) | 0.06 | -2.35 (-7.65, 2.95) | 0.38 | -2.40 (-7.70, 2.91) | 0.38 |
| P for trend |  | 0.08 |  | 0.07 |  | 0.61 |  | 0.58 |
| Ln (Hexanaldehyde, ng/mL) | 0.46 (-0.12, 1.05) | 0.12 | 0.46 (-0.12, 1.05) | 0.12 | 0.55 (-0.02, 1.12) | 0.06 | 0.52 (-0.05, 1.09) | 0.08 |
| Hexanaldehyde (quartiles) |  |  |  |  |  |  |  |  |
| Q1 | 1 (reference) |  | 1 (reference) |  | 1 (reference) | 0 | 1 (reference) |  |
| Q2 | 0.34 (-3.82, 4.50) | 0.87 | 0.40 (-3.76, 4.56) | 0.85 | 0.71 (-3.66, 5.07) | 0.75 | 0.62 (-3.76, 4.99) | 0.78 |
| Q3 | -2.17 (-6.26, 1.93) | 0.3 | -2.15 (-6.25, 1.95) | 0.30 | -2.57 (-6.85, 1.71) | 0.24 | -2.61 (-6.90, 1.67) | 0.23 |
| Q4 | -1.12 (-5.12, 2.89) | 0.59 | -0.99 (-5.00, 3.01) | 0.63 | -1.08 (-5.34, 3.18) | 0.62 | -1.12 (-5.40, 3.15) | 0.61 |
| P for trend |  | 0.38 |  | 0.41 |  | 0.35 |  | 0.35 |
| Ln (Heptanaldehyde, ng/mL | 2.69 (-4.80, 10.18) | 0.48 | 3.26 (-4.27, 10.80) | 0.40 | 4.85 (-2.93, 12.63) | 0.22 | 4.83 (-2.95, 12.60) | 0.22 |
| Heptanaldehyde (quartiles) |  |  |  |  |  |  |  |  |
| Q1 | 1 (reference) |  | 1 (reference) |  | 1 (reference) |  | 1 (reference) |  |
| Q2 | -0.93 (-5.05, 3.18) | 0.66 | -0.96 (-5.08, 3.16) | 0.65 | -1.53 (-5.84, 2.77) | 0.48 | -1.77 (-6.07, 2.54) | 0.42 |
| Q3 | 1.86 (-2.26, 5.98) | 0.38 | 1.99 (-2.14, 6.11) | 0.35 | 2.09 (-2.24, 6.42) | 0.35 | 2.12 (-2.21, 6.45) | 0.34 |
| Q4 | -0.82 (-5.06, 3.41) | 0.7 | -0.40 (-4.69, 3.90) | 0.86 | -0.48 (-4.99, 4.02) | 0.83 | -0.39 (-4.89, 4.10) | 0.86 |
| P for trend |  | 0.92 |  | 0.74 |  | 0.70 |  | 0.64 |
| Ln (Butyraldehyde, ng/mL) | 4.06 (-0.20, 8.31) | 0.06 | 4.29 (0.01, 8.57) | 0.05 | 3.76 (-0.83, 8.36) | 0.11 | 3.77 (-0.82, 8.37) | 0.11 |
| Butyraldehyde (quartiles) |  |  |  |  |  |  |  |  |
| Q1 | 1 (reference) |  | 1 (reference) |  | 1 (reference) |  | 1 (reference) |  |
| Q2 | 0.89 (-3.48, 5.26) | 0.69 | 0.98 (-3.39, 5.35) | 0.66 | -0.42 (-4.91, 4.07) | 0.85 | -0.26 (-4.76, 4.23) | 0.91 |
| Q3 | 2.89 (-1.31, 7.09) | 0.18 | 3.02 (-1.19, 7.24) | 0.16 | 2.60 (-1.75, 6.95) | 0.24 | 2.64 (-1.70, 6.99) | 0.23 |
| Q4 | 3.66 (-0.56, 7.89) | 0.09 | 3.90 (-0.35, 8.15) | 0.07 | 3.03 (-1.41, 7.48) | 0.18 | 3.02 (-1.44, 7.48) | 0.18 |
| P for trend |  | 0.05 |  | 0.04 |  | 0.08 |  | 0.09 |
| Ln (Benzaldehyde, ng/mL) | 0.05 (-0.75, 0.84) | 0.91 | 0.03 (-0.77, 0.83) | 0.94 | 0.08 (-0.72, 0.87) | 0.85 | 0.07 (-0.72, 0.87) | 0.86 |
| Benzaldehyde (quartiles) |  |  |  |  |  |  |  |  |
| Q1 | 1 (reference) |  | 1 (reference) |  | 1 (reference) |  | 1 (reference) |  |
| Q2 | -0.67 (-4.50, 3.16) | 0.73 | -0.63 (-4.47, 3.21) | 0.75 | 2.81 (-1.26, 6.87) | 0.18 | 2.83 (-1.24, 6.89) | 0.17 |
| Q3 | -3.01 (-6.93, 0.92) | 0.13 | -3.01 (-6.95, 0.92) | 0.13 | -0.35 (-4.50, 3.80) | 0.87 | -0.43 (-4.59, 3.72) | 0.84 |
| Q4 | 0.51 (-3.58, 4.59) | 0.81 | 0.41 (-3.69, 4.52) | 0.84 | 2.57 (-1.78, 6.92) | 0.25 | 2.68 (-1.67, 7.03) | 0.23 |
| P for trend |  | 0.79 |  | 0.75 |  | 0.5 |  | 0.48 |
| **Absolute neutrophil count** |  |  |  |  |  |  |  |  |
| Ln (Propanaldehyde, ng/mL) | 0.32 (0.15, 0.50) | <0.01 | 0.30 (0.13, 0.48) | <0.01 | 0.18 (-0.01, 0.38) | 0.07 | 0.19 (-0.00, 0.39) | 0.05 |
| Propanaldehyde (quartiles) |  |  |  |  |  |  |  |  |
| Q1 | 1 (reference) |  | 1 (reference) |  | 1 (reference) |  | 1 (reference) |  |
| Q2 | -0.17 (-0.64, 0.29) | 0.47 | -0.18 (-0.65, 0.29) | 0.45 | -0.25 (-0.73, 0.24) | 0.32 | -0.25 (-0.73, 0.23) | 0.31 |
| Q3 | 0.17 (-0.29, 0.63) | 0.48 | 0.17 (-0.29, 0.63) | 0.47 | -0.10 (-0.58, 0.38) | 0.68 | -0.15 (-0.63, 0.33) | 0.54 |
| Q4 | 0.54 (0.09, 0.99) | 0.02 | 0.49 (0.04, 0.94) | 0.03 | 0.11 (-0.38, 0.61) | 0.65 | 0.13 (-0.36, 0.63) | 0.6 |
| P for trend |  | 0.01 |  | 0.01 |  | 0.56 |  | 0.55 |
| Ln (Isopentanaldehyde, ng/mL) | 0.86 (0.55, 1.17) | <0.0001 | 0.81 (0.50, 1.11) | <0.0001 | 0.57 (0.13, 1.01) | 0.01 | 0.50 (-0.89, 1.88) | 0.48 |
| Isopentanaldehyde (quartiles) |  |  |  |  |  |  |  |  |
| Q1 | 1 (reference) |  | 1 (reference) |  | 1 (reference) |  | 1 (reference) |  |
| Q2 | -0.10 (-0.58, 0.38) | 0.68 | -0.03 (-0.50, 0.45) | 0.91 | -0.13 (-0.64, 0.37) | 0.6 | -0.18 (-0.68, 0.33) | 0.49 |
| Q3 | 0.21 (-0.26, 0.69) | 0.38 | 0.30 (-0.17, 0.77) | 0.21 | 0.11 (-0.39, 0.61) | 0.67 | 0.08 (-0.42, 0.58) | 0.76 |
| Q4 | 0.84 (0.38, 1.29) | 0 | 0.87 (0.42, 1.33) | 0 | 0.17 (-0.43, 0.76) | 0.59 | 0.11 (-0.48, 0.71) | 0.71 |
| P for trend |  | <0.0001 |  | <0.0001 |  | 0.42 |  | 0.51 |
| Ln (Hexanaldehyde, ng/mL) | 0.04 (-0.02, 0.11) | 0.19 | 0.04 (-0.02, 0.11) | 0.21 | 0.05 (-0.02, 0.11) | 0.15 | 0.04 (-0.02, 0.11) | 0.19 |
| Hexanaldehyde (quartiles) |  |  |  |  |  |  |  |  |
| Q1 | 1 (reference) |  | 1 (reference) |  | 1 (reference) |  | 1 (reference) |  |
| Q2 | -0.18 (-0.66, 0.29) | 0.45 | -0.19 (-0.66, 0.28) | 0.43 | -0.19 (-0.69, 0.30) | 0.45 | -0.24 (-0.74, 0.25) | 0.34 |
| Q3 | -0.31 (-0.78, 0.15) | 0.19 | -0.33 (-0.79, 0.14) | 0.17 | -0.31 (-0.79, 0.18) | 0.21 | -0.35 (-0.84, 0.13) | 0.15 |
| Q4 | -0.19 (-0.65, 0.27) | 0.41 | -0.23 (-0.68, 0.22) | 0.32 | -0.20 (-0.68, 0.28) | 0.41 | -0.26 (-0.75, 0.22) | 0.28 |
| P for trend |  | 0.37 |  | 0.28 |  | 0.38 |  | 0.26 |
| Ln (Heptanaldehyde, ng/mL) | 0.34 (-0.52, 1.19) | 0.44 | 0.15 (-0.70, 1.01) | 0.73 | 0.11 (-0.78, 0.99) | 0.82 | 0.13 (-0.75, 1.01) | 0.77 |
| Heptanaldehyde (quartiles) |  |  |  |  |  |  |  |  |
| Q1 | 1 (reference) |  | 1 (reference) |  | 1 (reference) |  | 1 (reference) |  |
| Q2 | -0.09 (-0.56, 0.38) | 0.71 | -0.09 (-0.55, 0.38) | 0.71 | -0.07 (-0.55, 0.42) | 0.79 | -0.11 (-0.59, 0.38) | 0.67 |
| Q3 | -0.31 (-0.78, 0.16) | 0.2 | -0.36 (-0.83, 0.10) | 0.13 | -0.37 (-0.86, 0.12) | 0.14 | -0.36 (-0.85, 0.13) | 0.15 |
| Q4 | 0.02 (-0.46, 0.51) | 0.92 | -0.09 (-0.58, 0.40) | 0.72 | -0.16 (-0.67, 0.35) | 0.55 | -0.15 (-0.66, 0.36) | 0.56 |
| P for trend |  | 0.85 |  | 0.47 |  | 0.32 |  | 0.37 |
| Ln (Butyraldehyde, ng/mL) | 0.19 (-0.30, 0.68) | 0.45 | 0.17 (-0.31, 0.66) | 0.49 | -0.17 (-0.69, 0.35) | 0.53 | -0.20 (-0.71, 0.32) | 0.46 |
| Butyraldehyde (quartiles) |  |  |  |  |  |  |  |  |
| Q1 | 1 (reference) |  | 1 (reference) |  | 1 (reference) |  | 1 (reference) |  |
| Q2 | -0.11 (-0.61, 0.39) | 0.68 | -0.11 (-0.61, 0.38) | 0.66 | -0.18 (-0.69, 0.33) | 0.49 | -0.15 (-0.66, 0.35) | 0.56 |
| Q3 | -0.04 (-0.52, 0.44) | 0.88 | -0.03 (-0.51, 0.45) | 0.9 | -0.15 (-0.64, 0.34) | 0.56 | -0.15 (-0.65, 0.34) | 0.54 |
| Q4 | 0.25 (-0.23, 0.73) | 0.31 | 0.24 (-0.24, 0.73) | 0.32 | 0.02 (-0.48, 0.53) | 0.93 | -0.03 (-0.53, 0.48) | 0.92 |
| P for trend |  | 0.23 |  | 0.23 |  | 0.85 |  | 0.96 |
| Ln (Benzaldehyde, ng/mL) | -0.03 (-0.12, 0.06) | 0.56 | -0.03 (-0.12, 0.06) | 0.52 | -0.03 (-0.12, 0.06) | 0.5 | -0.04 (-0.12, 0.05) | 0.44 |
| Benzaldehyde (quartiles) |  |  |  |  |  |  |  |  |
| Q1 | 1 (reference) |  | 1 (reference) |  | 1 (reference) |  | 1 (reference) |  |
| Q2 | -0.09 (-0.53, 0.35) | 0.68 | -0.13 (-0.56, 0.31) | 0.57 | 0.09 (-0.37, 0.55) | 0.71 | 0.08 (-0.38, 0.54) | 0.74 |
| Q3 | -0.27 (-0.72, 0.18) | 0.23 | -0.31 (-0.76, 0.14) | 0.18 | -0.14 (-0.61, 0.34) | 0.57 | -0.13 (-0.60, 0.33) | 0.58 |
| Q4 | -0.00 (-0.47, 0.47) | 1 | -0.01 (-0.48, 0.45) | 0.95 | 0.12 (-0.37, 0.62) | 0.63 | 0.11 (-0.38, 0.60) | 0.66 |
| P for trend |  | 0.72 |  | 0.67 |  | 0.88 |  | 0.91 |
| **Lymphocyte count** |  |  |  |  |  |  |  |  |
| Ln (Propanaldehyde, ng/mL) | 0.12 (0.06, 0.17) | <0.001 | 0.11 (0.06, 0.16) | <0.001 | 0.10 (0.04, 0.16) | <0.01 | **0.10 (0.04, 0.16)** | **<0.01** |
| Propanaldehyde (quartiles) |  |  |  |  |  |  |  |  |
| Q1 | 1 (reference) |  | 1 (reference) |  | 1 (reference) |  | 1 (reference) |  |
| Q2 | -0.03 (-0.17, 0.12) | 0.73 | -0.02 (-0.16, 0.13) | 0.81 | -0.05 (-0.21, 0.10) | 0.5 | -0.05 (-0.21, 0.10) | 0.5 |
| Q3 | 0.04 (-0.11, 0.18) | 0.61 | 0.05 (-0.09, 0.19) | 0.47 | 0.00 (-0.15, 0.16) | 0.96 | -0.01 (-0.16, 0.15) | 0.92 |
| Q4 | 0.31 (0.16, 0.45) | <0.001 | 0.29 (0.16, 0.43) | <0.001 | 0.23 (0.07, 0.39) | <0.01 | 0.23 (0.08, 0.39) | <0.01 |
| P for trend |  | <0.001 |  | <0.001 |  | <0.01 |  | <0.01 |
| Ln (Isopentanaldehyde, ng/mL) | 0.22 (0.12, 0.32) | <0.001 | 0.20 (0.10, 0.29) | <0.001 | 0.23 (0.09, 0.37) | <0.01 | **0.23 (0.08, 0.37)** | **<0.01** |
| Isopentanaldehyde (quartiles) |  |  |  |  |  |  |  |  |
| Q1 | 1 (reference) |  | 1 (reference) |  | 1 (reference) |  | 1 (reference) |  |
| Q2 | -0.15 (-0.30, 0.01) | 0.06 | -0.10 (-0.25, 0.05) | 0.19 | -0.12 (-0.28, 0.05) | 0.16 | -0.13 (-0.29, 0.03) | 0.12 |
| Q3 | -0.03 (-0.18, 0.12) | 0.71 | 0.03 (-0.12, 0.18) | 0.68 | -0.01 (-0.17, 0.15) | 0.93 | -0.01 (-0.17, 0.15) | 0.92 |
| Q4 | 0.13 (-0.01, 0.28) | 0.07 | 0.17 (0.03, 0.31) | 0.02 | 0.08 (-0.11, 0.27) | 0.43 | 0.06 (-0.13, 0.26) | 0.52 |
| P for trend |  | 0.01 |  | <0.01 |  | 0.31 |  | 0.35 |
| Ln (Hexanaldehyde, ng/mL) | 0.02 (-0.00, 0.04) | 0.06 | 0.02 (-0.00, 0.04) | 0.08 | 0.02 (-0.00, 0.04) | 0.12 | 0.02 (-0.00, 0.04) | 0.11 |
| Hexanaldehyde (quartiles) |  |  |  |  |  |  |  |  |
| Q1 | 1 (reference) |  | 1 (reference) |  | 1 (reference) |  | 1 (reference) |  |
| Q2 | 0.01 (-0.14, 0.16) | 0.87 | 0.01 (-0.13, 0.16) | 0.85 | 0.00 (-0.16, 0.16) | 0.99 | -0.01 (-0.17, 0.15) | 0.89 |
| Q3 | 0.06 (-0.09, 0.21) | 0.42 | 0.05 (-0.09, 0.20) | 0.48 | 0.09 (-0.07, 0.24) | 0.27 | 0.08 (-0.08, 0.23) | 0.34 |
| Q4 | 0.16 (0.02, 0.31) | 0.03 | 0.15 (0.01, 0.29) | 0.04 | 0.14 (-0.01, 0.30) | 0.07 | 0.13 (-0.03, 0.28) | 0.11 |
| P for trend |  | 0.02 |  | 0.03 |  | 0.04 |  | 0.06 |
| Ln (Heptanaldehyde, ng/mL) | 0.26 (-0.01, 0.53) | 0.06 | 0.17 (-0.09, 0.44) | 0.20 | 0.15 (-0.14, 0.43) | 0.31 | 0.16 (-0.13, 0.44) | 0.28 |
| Heptanaldehyde (quartiles) |  |  |  |  |  |  |  |  |
| Q1 | 1 (reference) |  | 1 (reference) |  | 1 (reference) |  | 1 (reference) |  |
| Q2 | 0.06 (-0.09, 0.21) | 0.44 | 0.06 (-0.09, 0.20) | 0.45 | 0.06 (-0.09, 0.22) | 0.43 | 0.06 (-0.10, 0.22) | 0.45 |
| Q3 | 0.03 (-0.12, 0.17) | 0.74 | -0.00 (-0.15, 0.14) | 0.96 | 0.02 (-0.14, 0.18) | 0.83 | 0.02 (-0.14, 0.18) | 0.81 |
| Q4 | 0.12 (-0.03, 0.27) | 0.13 | 0.08 (-0.07, 0.23) | 0.28 | 0.07 (-0.10, 0.23) | 0.41 | 0.07 (-0.10, 0.23) | 0.42 |
| P for trend |  | 0.2 |  | 0.46 |  | 0.58 |  | 0.57 |
| Ln (Butyraldehyde, ng/mL) | 0.15 (-0.02, 0.31) | 0.09 | 0.20 (0.05, 0.35) | 0.01 | 0.16 (-0.01, 0.32) | 0.07 | **0.18 (0.03, 0.34)** | **0.02** |
| Butyraldehyde (quartiles) |  |  |  |  |  |  |  |  |
| Q1 | 1 (reference) |  | 1 (reference) |  | 1 (reference) |  | 1 (reference) |  |
| Q2 | 0.13 (-0.03, 0.29) | 0.11 | 0.13 (-0.02, 0.29) | 0.09 | 0.07 (-0.09, 0.23) | 0.40 | 0.07 (-0.09, 0.23) | 0.39 |
| Q3 | 0.12 (-0.03, 0.27) | 0.12 | 0.14 (-0.01, 0.29) | 0.07 | 0.14 (-0.02, 0.30) | 0.09 | 0.14 (-0.02, 0.29) | 0.09 |
| Q4 | 0.25 (0.10, 0.40) | <0.01 | 0.27 (0.12, 0.42) | <0.01 | 0.24 (0.07, 0.40) | <0.01 | 0.22 (0.06, 0.38) | 0.01 |
| P for trend |  | <0.01 |  | <0.01 |  | <0.01 |  | <0.01 |
| Ln (Benzaldehyde, ng/mL) | -0.00 (-0.03, 0.03) | 0.88 | -0.01 (-0.03, 0.02) | 0.64 | -0.01 (-0.03, 0.02) | 0.71 | -0.01 (-0.04, 0.02) | 0.65 |
| Benzaldehyde (quartiles) |  |  |  |  |  |  |  |  |
| Q1 | 1 (reference) |  | 1 (reference) |  | 1 (reference) |  | 1 (reference) |  |
| Q2 | 0.07 (-0.07, 0.21) | 0.34 | 0.05 (-0.09, 0.18) | 0.50 | 0.10 (-0.05, 0.25) | 0.19 | 0.10 (-0.05, 0.24) | 0.20 |
| Q3 | 0.03 (-0.11, 0.17) | 0.68 | 0.00 (-0.14, 0.14) | 0.98 | 0.05 (-0.10, 0.20) | 0.53 | 0.05 (-0.10, 0.20) | 0.50 |
| Q4 | 0.05 (-0.10, 0.20) | 0.51 | 0.03 (-0.12, 0.17) | 0.71 | 0.05 (-0.11, 0.21) | 0.52 | 0.04 (-0.11, 0.20) | 0.58 |
| P for trend |  | 0.61 |  | 0.86 |  | 0.6 |  | 0.64 |

Model 1: adjusted for age and sex. Model 2: Model 1 plus race/ethnicity, family PIR, education level, serum cotinine level, BMI category, and past-year alcohol consumption. Model 3: Model 2 plus diabetes and hypertension. Bold values indicate statistical significance (P < 0.05). PIR poverty income ratio, OR odds ratio, CI confidence interval.

Supplementary S4: Association between serum levels of aldehydes and markers of oxidative stress in a multivariate linear regression model.

| **Exposure** | **Non-adjusted** | ***P*** | **Model 1** | ***P*** | **Model 2** | ***P*** | **Model 3** | ***P*** |
| --- | --- | --- | --- | --- | --- | --- | --- | --- |
|  | OR (95% CI) |  | OR (95% CI) |  | OR (95% CI) |  | OR (95% CI) |  |
| **Bilirubin** |  |  |  |  |  |  |  |  |
| Ln (Propanaldehyde, ng/mL) | 0.01 (-0.02, 0.03) | 0.63 | 0.00 (-0.02, 0.02) | 0.98 | 0.01 (-0.01, 0.04) | 0.35 | 0.01 (-0.01, 0.04) | 0.35 |
| Propanaldehyde (quartiles) |  |  |  |  |  |  |  |  |
| Q1 | 1 (reference) |  | 1 (reference) |  | 1 (reference) | 1 (reference) | 1 (reference) |  |
| Q2 | 0.01 (-0.05, 0.07) | 0.74 | -0.00 (-0.06, 0.05) | 0.94 | -0.01 (-0.07, 0.05) | 0.82 | -0.01 (-0.07, 0.05) | 0.81 |
| Q3 | 0.01 (-0.05, 0.07) | 0.75 | -0.01 (-0.06, 0.05) | 0.84 | 0.03 (-0.03, 0.09) | 0.29 | 0.03 (-0.03, 0.09) | 0.30 |
| Q4 | 0.03 (-0.03, 0.09) | 0.28 | 0.02 (-0.04, 0.07) | 0.56 | 0.04 (-0.02, 0.10) | 0.19 | 0.04 (-0.02, 0.10) | 0.19 |
| *P* for trend |  | 0.3 |  | 0.59 |  | 0.11 |  | 0.10 |
| Ln (Isopentanaldehyde, ng/mL) | -0.04 (-0.08, 0.00) | 0.06 | -0.05 (-0.09, -0.01) | 0.01 | 0.00 (-0.06, 0.06) | 0.99 | 0.00 (-0.06, 0.06) | 0.99 |
| Isopentanaldehyde (quartiles) |  |  |  |  |  |  |  |  |
| Q1 | 1 (reference) |  | 1 (reference |  | 1 (reference |  | 1 (reference |  |
| Q2 | -0.04 (-0.10, 0.02) | 0.15 | -0.06 (-0.12, 0.00) | 0.05 | -0.07 (-0.13, -0.01) | 0.03 | -0.07 (-0.13, -0.01) | 0.03 |
| Q3 | 0.02 (-0.04, 0.08) | 0.52 | -0.00 (-0.06, 0.05) | 0.89 | -0.01 (-0.07, 0.06) | 0.83 | -0.01 (-0.07, 0.06) | 0.8 |
| Q4 | -0.06 (-0.12, -0.00) | 0.04 | -0.09 (-0.14, -0.03) | < 0.01 | -0.04 (-0.12, 0.03) | 0.25 | -0.04 (-0.12, 0.03) | 0.25 |
| P for trend |  | 0.17 |  | 0.02 |  | 0.67 |  | 0.67 |
| Ln (Hexanaldehyde, ng/mL) | 0.00 (-0.01, 0.01) | 0.99 | 0.00 (-0.01, 0.01) | 0.76 | 0.00 (-0.01, 0.01) | 0.91 | 0.00 (-0.01, 0.01) | 0.95 |
| Hexanaldehyde (quartiles) |  |  |  |  |  |  |  |  |
| Q1 | 1 (reference) |  | 1 (reference) | 0 | 1 (reference) | 0 | 1 (reference) |  |
| Q2 | 0.00 (-0.06, 0.06) | 0.97 | -0.00 (-0.06, 0.05) | 0.88 | -0.01 (-0.08, 0.05) | 0.66 | -0.01 (-0.08, 0.05) | 0.66 |
| Q3 | -0.01 (-0.07, 0.05) | 0.75 | -0.01 (-0.06, 0.05) | 0.81 | -0.02 (-0.08, 0.04) | 0.49 | -0.02 (-0.08, 0.04) | 0.51 |
| Q4 | 0.04 (-0.02, 0.10) | 0.17 | 0.04 (-0.02, 0.09) | 0.2 | 0.03 (-0.03, 0.09) | 0.35 | 0.03 (-0.03, 0.09) | 0.34 |
| P for trend |  | 0.21 |  | 0.21 |  | 0.37 |  | 0.36 |
| Ln (Heptanaldehyde, ng/mL) | -0.05 (-0.15, 0.06) | 0.38 | -0.06 (-0.16, 0.05) | 0.27 | -0.07 (-0.18, 0.05) | 0.25 | -0.07 (-0.18, 0.04) | 0.24 |
| Heptanaldehyde (quartiles) |  |  |  |  |  |  |  |  |
| Q1 | 1 (reference) |  | 1 (reference) |  | 1 (reference) |  | 1 (reference) |  |
| Q2 | 0.01 (-0.05, 0.07) | 0.81 | 0.01 (-0.05, 0.07) | 0.7 | 0.02 (-0.04, 0.08) | 0.51 | 0.02 (-0.04, 0.08) | 0.52 |
| Q3 | -0.03 (-0.09, 0.03) | 0.34 | -0.03 (-0.08, 0.03) | 0.37 | -0.03 (-0.09, 0.04) | 0.43 | -0.03 (-0.09, 0.04) | 0.43 |
| Q4 | -0.01 (-0.07, 0.05) | 0.77 | -0.03 (-0.09, 0.03) | 0.29 | -0.04 (-0.10, 0.03) | 0.25 | -0.04 (-0.10, 0.03) | 0.26 |
| P for trend |  | 0.48 |  | 0.14 |  | 0.11 |  | 0.11 |
| Ln (Butyraldehyde, ng/mL) | -0.00 (-0.06, 0.06) | 0.99 | -0.02 (-0.08, 0.04) | 0.46 | 0.02 (-0.05, 0.08) | 0.64 | 0.02 (-0.05, 0.08) | 0.63 |
| Butyraldehyde (quartiles) |  |  |  |  |  |  |  |  |
| Q1 | 1 (reference) |  | 1 (reference) |  | 1 (reference) |  | 1 (reference) |  |
| Q2 | -0.01 (-0.07, 0.05 | 0.79 | -0.02 (-0.08, 0.04) | 0.58 | -0.01 (-0.08, 0.05) | 0.72 | -0.01 (-0.08, 0.05) | 0.74 |
| Q3 | -0.05 (-0.11, 0.01 | 0.13 | -0.06 (-0.12, -0.00) | 0.04 | -0.06 (-0.12, 0.01) | 0.08 | -0.05 (-0.12, 0.01) | 0.08 |
| Q4 | 0.01 (-0.05, 0.07 | 0.79 | -0.02 (-0.07, 0.04) | 0.6 | 0.02 (-0.05, 0.08) | 0.60 | 0.02 (-0.05, 0.08) | 0.58 |
| P for trend |  | 1 |  | 0.4 |  | 0.88 |  | 0.87 |
| Ln (Benzaldehyde, ng/mL) | -0.00 (-0.01, 0.01 | 0.9 | 0.00 (-0.01, 0.01 | 0.61 | 0.00 (-0.01, 0.01) | 0.83 | 0.00 (-0.01, 0.01) | 0.82 |
| Benzaldehyde (quartiles) |  |  |  |  |  |  |  |  |
| Q1 | 1 (reference) |  | 1 (reference) |  | 1 (reference) |  | 1 (reference) |  |
| Q2 | 0.03 (-0.03, 0.08) | 0.3 | 0.03 (-0.02, 0.09) | 0.2 | 0.02 (-0.04, 0.08) | 0.48 | 0.02 (-0.04, 0.08) | 0.48 |
| Q3 | 0.03 (-0.02, 0.09) | 0.24 | 0.05 (-0.01, 0.10) | 0.09 | 0.02 (-0.04, 0.08) | 0.47 | 0.02 (-0.04, 0.08) | 0.48 |
| Q4 | 0.01 (-0.05, 0.07) | 0.68 | 0.03 (-0.03, 0.09) | 0.29 | 0.01 (-0.05, 0.07) | 0.82 | 0.01 (-0.05, 0.07) | 0.79 |
| P for trend |  | 0.58 |  | 0.21 |  | 0.75 |  | 0.73 |
| **Albumin** |  |  |  |  |  |  |  |  |
| Ln (Propanaldehyde, ng/mL) | 0.02 (-0.01, 0.04 | 0.13 | 0.01 (-0.01, 0.03) | 0.44 | 0.01 (-0.01, 0.04) | 0.38 | 0.01 (-0.01, 0.04) | 0.39 |
| Propanaldehyde (quartiles) |  |  |  |  |  |  |  |  |
| Q1 | 1 (reference) |  | 1 (reference) |  | 1 (reference) |  | 1 (reference) |  |
| Q2 | 0.08 (0.02, 0.14) | 0.01 | 0.07 (0.00, 0.13) | 0.04 | 0.07 (0.01, 0.14) | 0.02 | 0.07 (0.01, 0.14) | 0.02 |
| Q3 | 0.03 (-0.03, 0.10) | 0.31 | 0.02 (-0.04, 0.08) | 0.60 | 0.02 (-0.04, 0.08) | 0.56 | 0.02 (-0.04, 0.08) | 0.57 |
| Q4 | 0.06 (0.00, 0.13) | 0.04 | 0.04 (-0.02, 0.10) | 0.20 | 0.04 (-0.02, 0.11) | 0.22 | 0.04 (-0.02, 0.11) | 0.21 |
| P for trend |  | 0.15 |  | 0.51 |  | 0.50 |  | 0.50 |
| Ln (Isopentanaldehyde, ng/mL) | 0.02 (-0.02, 0.06) | 0.40 | -0.01 (-0.05, 0.03) | 0.77 | 0.03 (-0.03, 0.09) | 0.29 | 0.03 (-0.03, 0.09) | 0.30 |
| Isopentanaldehyde (quartiles) |  |  |  |  |  |  |  |  |
| Q1 | 1 (reference) |  | 1 (reference) |  | 1 (reference) |  | 1 (reference) |  |
| Q2 | 0.06 (-0.00, 0.13) | 0.06 | 0.06 (0.00, 0.13) | 0.05 | 0.05 (-0.01, 0.12) | 0.11 | 0.05 (-0.01, 0.12) | 0.11 |
| Q3 | 0.09 (0.03, 0.16) | 0.01 | 0.08 (0.02, 0.14) | 0.01 | 0.09 (0.02, 0.16) | 0.01 | 0.09 (0.02, 0.15) | 0.01 |
| Q4 | 0.05 (-0.01, 0.12) | 0.10 | 0.03 (-0.03, 0.09) | 0.31 | 0.08 (0.00, 0.16) | 0.04 | 0.08 (0.00, 0.16) | 0.04 |
| P for trend |  | 0.10 |  | 0.38 |  | 0.01 |  | 0.01 |
| Ln (Hexanaldehyde, ng/mL) | -0.00 (-0.01, 0.01) | 0.85 | -0.00 (-0.01, 0.01) | 1 | 0.00 (-0.01, 0.01) | 0.89 | 0.00 (-0.01, 0.01) | 0.95 |
| Hexanaldehyde (quartiles) |  |  |  |  |  |  |  |  |
| Q1 | 1 (reference) |  | 1 (reference) |  | 1 (reference) |  | 1 (reference) |  |
| Q2 | 0.04 (-0.02, 0.11) | 0.19 | 0.04 (-0.03, 0.10) | 0.26 | 0.04 (-0.02, 0.11) | 0.22 | 0.04 (-0.02, 0.11) | 0.22 |
| Q3 | 0.06 (-0.01, 0.12) | 0.07 | 0.06 (-0.00, 0.12) | 0.06 | 0.07 (0.00, 0.13) | 0.04 | 0.07 (0.00, 0.13) | 0.04 |
| Q4 | 0.04 (-0.02, 0.11) | 0.18 | 0.03 (-0.03, 0.09) | 0.30 | 0.04 (-0.03, 0.10) | 0.26 | 0.04 (-0.03, 0.10) | 0.24 |
| P for trend |  | 0.17 |  | 0.26 |  | 0.21 |  | 0.20 |
| Ln (Heptanaldehyde, ng/mL) | 0.07 (-0.05, 0.18) | 0.27 | 0.02 (-0.09, 0.13) | 0.75 | 0.00 (-0.11, 0.12) | 0.94 | 0.00 (-0.11, 0.12) | 0.95 |
| Heptanaldehyde (quartiles) |  |  |  |  |  |  |  |  |
| Q1 | 1 (reference) |  | 1 (reference) |  | 1 (reference) |  | 1 (reference) |  |
| Q2 | 0.05 (-0.01, 0.12) | 0.10 | 0.06 (-0.00, 0.12) | 0.06 | 0.07 (0.00, 0.13) | 0.04 | 0.07 (0.00, 0.13) | 0.04 |
| Q3 | 0.05 (-0.01, 0.11) | 0.13 | 0.04 (-0.02, 0.10) | 0.18 | 0.03 (-0.03, 0.10) | 0.29 | 0.03 (-0.03, 0.10) | 0.29 |
| Q4 | 0.08 (0.01, 0.15) | 0.02 | 0.03 (-0.03, 0.10) | 0.31 | 0.03 (-0.04, 0.10) | 0.39 | 0.03 (-0.04, 0.10) | 0.38 |
| P for trend |  | 0.03 |  | 0.48 |  | 0.70 |  | 0.67 |
| Ln (Butyraldehyde, ng/mL) | 0.06 (-0.00, 0.13) | 0.06 | 0.04 (-0.02, 0.10) | 0.24 | 0.04 (-0.03, 0.11) | 0.29 | 0.04 (-0.03, 0.11) | 0.28 |
| Butyraldehyde (quartiles) |  |  |  |  |  |  |  |  |
| Q1 | 1 (reference) |  | 1 (reference) |  | 1 (reference) |  | 1 (reference) |  |
| Q2 | 0.03 (-0.03, 0.10) | 0.33 | 0.02 (-0.04, 0.09) | 0.47 | 0.04 (-0.03, 0.11) | 0.23 | 0.04 (-0.02, 0.11) | 0.22 |
| Q3 | 0.10 (0.04, 0.17) | < 0.01 | 0.09 (0.02, 0.15) | 0.01 | 0.08 (0.02, 0.15) | 0.01 | 0.08 (0.02, 0.15) | 0.01 |
| Q4 | 0.09 (0.03, 0.16) | < 0.01 | 0.07 (0.01, 0.13) | 0.03 | 0.07 (0.00, 0.13) | 0.04 | 0.07 (0.00, 0.13) | 0.04 |
| P for trend |  | < 0.01 |  | 0.01 |  | 0.02 |  | 0.02 |
| Ln (Benzaldehyde, ng/mL) | 0.00 (-0.01, 0.01) | 0.77 | 0.01 (-0.01, 0.02) | 0.37 | 0.00 (-0.01, 0.02) | 0.47 | 0.00 (-0.01, 0.02) | 0.46 |
| Benzaldehyde (quartiles) |  |  |  |  |  |  |  |  |
| Q1 | 1 (reference) |  | 1 (reference) |  | 1 (reference) |  | 1 (reference) |  |
| Q2 | 0.05 (-0.01, 0.11 | 0.08 | 0.05 (-0.00, 0.11) | 0.07 | 0.02 (-0.04, 0.08) | 0.45 | 0.02 (-0.04, 0.08) | 0.44 |
| Q3 | 0.07 (0.01, 0.13 | 0.03 | 0.08 (0.02, 0.13) | 0.01 | 0.05 (-0.01, 0.11) | 0.1 | 0.05 (-0.01, 0.11) | 0.11 |
| Q4 | 0.02 (-0.04, 0.09 | 0.48 | 0.04 (-0.02, 0.10) | 0.19 | 0.01 (-0.06, 0.07) | 0.78 | 0.01 (-0.05, 0.08) | 0.75 |
| P for trend |  | 0.3 |  | 0.09 |  | 0.5 |  | 0.49 |
| **Iron** |  |  |  |  |  |  |  |  |
| Ln (Propanaldehyde, ng/mL) | 4.43 (1.73, 7.13) | < 0.01 | 3.92 (1.24, 6.59) | < 0.01 | 3.27 (0.26, 6.28) | 0.03 | **3.25 (0.24, 6.27)** | **0.03** |
| Propanaldehyde (quartiles) |  |  |  |  |  |  |  |  |
| Q1 | 1 (reference) |  | 1 (reference) |  | 1 (reference) |  | 1 (reference) |  |
| Q2 | 7.04 (-0.11, 14.19) | 0.05 | 6.00 (-1.07, 13.06) | 0.1 | 5.37 (-2.12, 12.87) | 0.16 | 5.32 (-2.18, 12.82 | 0.16 |
| Q3 | 11.49 (4.44, 18.54) | < 0.01 | 10.23 (3.26, 17.20) | < 0.01 | 10.87 (3.40, 18.34) | < 0.01 | 10.85 (3.36, 18.34 | 0 |
| Q4 | 12.33 (5.42, 19.24) | < 0.01 | 10.92 (4.08, 17.77) | < 0.01 | 9.86 (2.22, 17.50) | 0.01 | 9.88 (2.23, 17.53 | 0.01 |
| P for trend |  | < 0.01 |  | < 0.01 |  | < 0.01 |  |  |
| Ln (Isopentanaldehyde, ng/mL) | 2.94 (-1.85, 7.74) | 0.23 | 1.84 (-2.92, 6.60 | 0.45 | -0.48 (-7.40, 6.44) | 0.89 | -0.52 (-7.44, 6.41 | 0.88 |
| Isopentanaldehyde (quartiles) |  |  |  |  |  |  |  |  |
| Q1 | 1 (reference) |  | 1 (reference) |  | 1 (reference) |  | 1 (reference) |  |
| Q2 | 7.29 (-0.08, 14.66 | 0.05 | 6.47 (-0.82, 13.77 | 0.08 | 5.71 (-2.13, 13.55) | 0.15 | 5.72 (-2.14, 13.58) | 0.15 |
| Q3 | 6.49 (-0.78, 13.77 | 0.08 | 4.90 (-2.34, 12.14 | 0.18 | 4.26 (-3.54, 12.06) | 0.28 | 4.04 (-3.77, 11.86) | 0.31 |
| Q4 | 6.87 (-0.15, 13.88 | 0.06 | 4.82 (-2.15, 11.79 | 0.18 | 2.82 (-6.48, 12.13) | 0.55 | 2.92 (-6.40, 12.24) | 0.54 |
| P for trend |  | 0.1 |  | 0.31 |  | 0.55 |  | 0.56 |
| Ln (Hexanaldehyde, ng/mL) | -0.08 (-1.11, 0.94 | 0.87 | 0.01 (-0.99, 1.02 | 0.98 | 0.07 (-0.94, 1.07 | 0.9 | 0.03 (-0.99, 1.04) | 0.96 |
| Hexanaldehyde (quartiles) |  |  |  |  |  |  |  |  |
| Q1 | 1 (reference) |  | 1 (reference) |  | 1 (reference) |  | 1 (reference) |  |
| Q2 | 8.69 (1.47, 15.92) | 0.02 | 8.18 (1.06, 15.29) | 0.02 | 7.59 (-0.06, 15.23) | 0.05 | 7.65 (-0.01, 15.32) | 0.05 |
| Q3 | 9.57 (2.46, 16.69) | 0.01 | 9.75 (2.74, 16.75) | 0.01 | 8.51 (1.02, 16.00) | 0.03 | 8.62 (1.12, 16.13) | 0.02 |
| Q4 | 9.89 (2.93, 16.84) | 0.01 | 9.40 (2.55, 16.26) | 0.01 | 10.08 (2.62, 17.53) | 0.01 | 10.28 (2.79, 17.77) | 0.01 |
| P for trend |  | 0.01 |  | 0.01 |  | 0.01 |  | 0.01 |
| Ln (Heptanaldehyde, ng/mL) | 5.02 (-8.06, 18.10) | 0.45 | 3.49 (-9.47, 16.45) | 0.6 | -2.01 (-15.70, 11.67) | 0.77 | -2.11 (-15.81, 11.58) | 0.76 |
| Heptanaldehyde (quartiles) |  |  |  |  |  |  |  |  |
| Q1 | 1 (reference) |  | 1 (reference) |  | 1 (reference) |  | 1 (reference) |  |
| Q2 | 2.50 (-4.69, 9.69) | 0.50 | 2.85 (-4.24, 9.94) | 0.43 | 1.21 (-6.37, 8.79) | 0.75 | 1.00 (-6.60, 8.59) | 0.80 |
| Q3 | 3.36 (-3.83, 10.55) | 0.36 | 3.40 (-3.70, 10.50) | 0.35 | 1.28 (-6.35, 8.92) | 0.74 | 1.30 (-6.33, 8.94) | 0.74 |
| Q4 | 6.83 (-0.57, 14.23) | 0.07 | 4.59 (-2.79, 11.97) | 0.22 | 1.21 (-6.72, 9.14) | 0.77 | 1.32 (-6.62, 9.25) | 0.74 |
| P for trend |  | 0.07 |  | 0.23 |  | 0.78 |  | 0.74 |
| Ln (Butyraldehyde, ng/mL) | 8.03 (-0.05, 16.10) | 0.05 | 6.49 (-0.87, 13.85) | 0.08 | 7.93 (-0.14, 16.00) | 0.05 | **8.40 (0.97, 15.83)** | **0.03** |
| Butyraldehyde (quartiles) |  |  |  |  |  |  |  |  |
| Q1 | 1 (reference) |  | 1 (reference) |  | 1 (reference) |  | 1 (reference) |  |
| Q2 | -0.28 (-7.89, 7.33) | 0.94 | -1.00 (-8.51, 6.50) | 0.79 | 0.34 (-7.55, 8.23) | 0.93 | 0.50 (-7.40, 8.40) | 0.90 |
| Q3 | 7.51 (0.19, 14.83) | 0.04 | 6.20 (-1.03, 13.44) | 0.09 | 5.68 (-1.97, 13.32) | 0.15 | 5.76 (-1.89, 13.40) | 0.14 |
| Q4 | 7.38 (0.03, 14.74) | 0.05 | 5.37 (-1.93, 12.67) | 0.15 | 4.19 (-3.63, 12.00) | 0.29 | 4.29 (-3.55, 12.13) | 0.28 |
| P for trend |  | 0.01 |  | 0.04 |  | 0.15 |  | 0.15 |
| Ln (Benzaldehyde, ng/mL) | 1.40 (0.02, 2.78) | 0.05 | 1.72 (0.36, 3.09) | 0.01 | 1.39 (-0.00, 2.78) | 0.05 | **1.39 (0.00, 2.78)** | **< 0.05** |
| Benzaldehyde (quartiles) |  |  |  |  |  |  |  |  |
| Q1 | 1 (reference) |  | 1 (reference) |  | 1 (reference) |  | 1 (reference) |  |
| Q2 | 3.97 (-2.69, 10.62) | 0.24 | 4.35 (-2.19, 10.89) | 0.19 | 1.74 (-5.35, 8.84) | 0.63 | 1.80 (-5.30, 8.90) | 0.62 |
| Q3 | 7.10 (0.28, 13.91) | 0.04 | 8.18 (1.48, 14.89) | 0.02 | 5.79 (-1.46, 13.04) | 0.12 | 5.66 (-1.59, 12.92) | 0.13 |
| Q4 | 12.72 (5.63, 19.82) | < 0.01 | 14.38 (7.38, 21.37) | <0.0001 | 12.69 (5.10, 20.28) | < 0.001 | 12.88 (5.28, 20.48) | < 0.001 |
| P for trend |  | < 0.01 |  | <0.0001 |  | < 0.001 |  | < 0.001 |

Model 1: adjusted for age and sex. Model 2: Model 1 plus race/ethnicity, family PIR, education level, serum cotinine level, BMI category, and past-year alcohol consumption. Model 3: Model 2 plus diabetes and hypertension. Bold values indicate statistical significance (P < 0.05). PIR poverty income ratio, OR odds ratio, CI confidence interval.

Supplementary S5. Interacting effects of sex and isopentanaldehyde on lymphocyte count. The models were adjusted for age, sex, race/ethnicity, PIR, education level, serum cotinine level, BMI, alcohol use, diabetes, and hypertension.


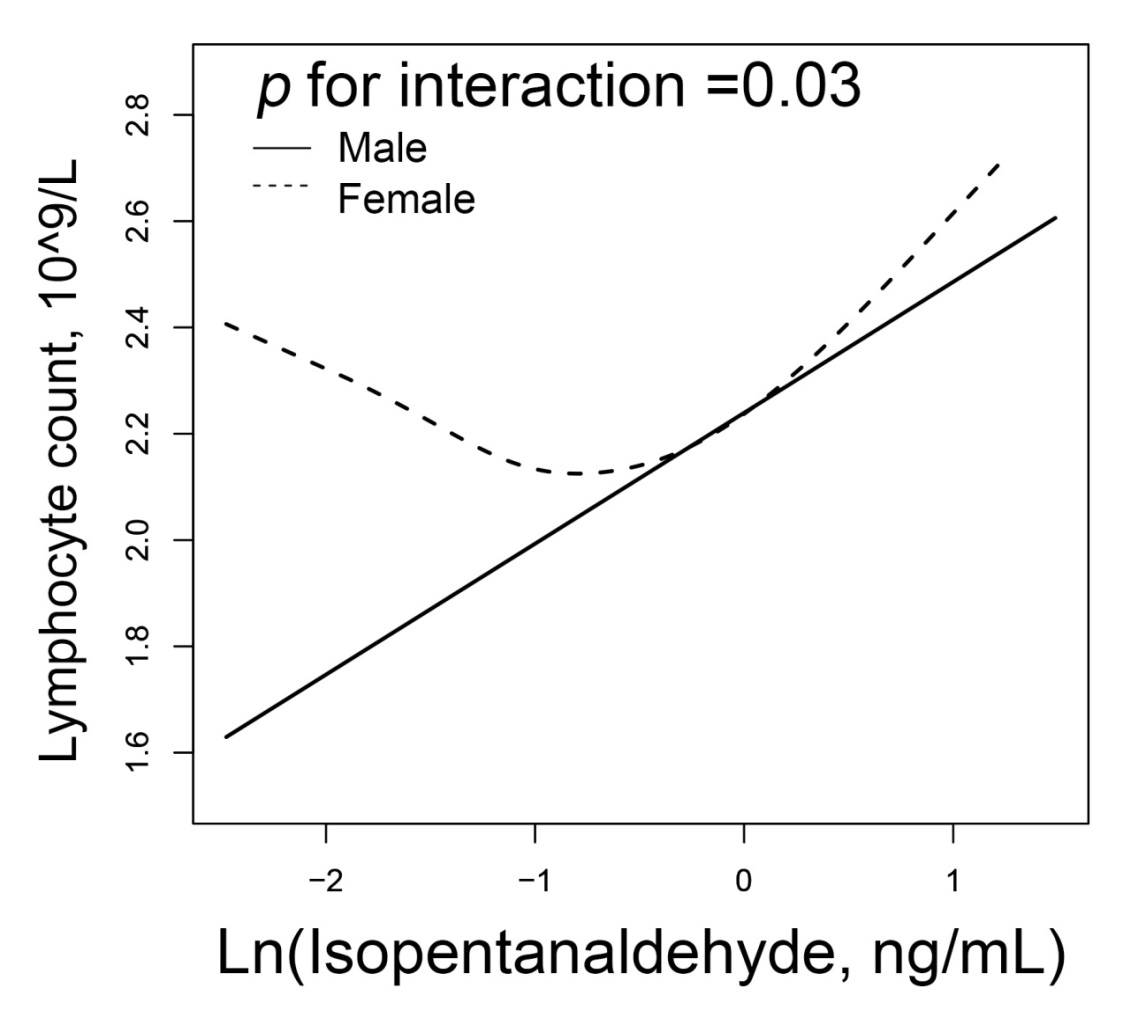


Supplementary S6. The WQS model for calculating the coefficient of the mixture index. The models were adjusted for age, sex, race/ethnicity, PIR, education level, serum cotinine level, BMI, alcohol use, diabetes, and hypertension.

|  | **Beta coefficient** | **95% CI** | ***p*** |
| --- | --- | --- | --- |
| **Alkaline phosphatase** | -0.02 | (-1.17,1.12) | 0.96 |
| **Bilirubin** | 0.009 | (-0.004, 0.02) | 0.18 |
| **Absolute neutrophil count** | -0.18 | (0.19, 0.93) | 0.35 |
| **Lymphocyte count** | 0.026 | (-0.01,0.06) | 0.16 |
| **Albumin** | 0.015 | (0.0007,0.03) | 0.03 |
| **Iron** | 3.45 | (1.63,5.27) | <0.001 |

Supplementary S7. Joint effect of the aldehyde mixture on outcomes by using the Bayesian kernel machine regression (BKMR) model. The models were adjusted for age, sex, race/ethnicity, PIR, education level, serum cotinine level, BMI, alcohol use, diabetes, and hypertension. (a) Overall effect of the mixture on the ALP level when all exposures are at a particular quantile to the median. (b) Overall effect of the mixture on the ANC when all exposures are at a particular quantile to the median. (c) Overall effect of the mixture on the serum bilirubin level when all exposures are at a particular quantile to the median.


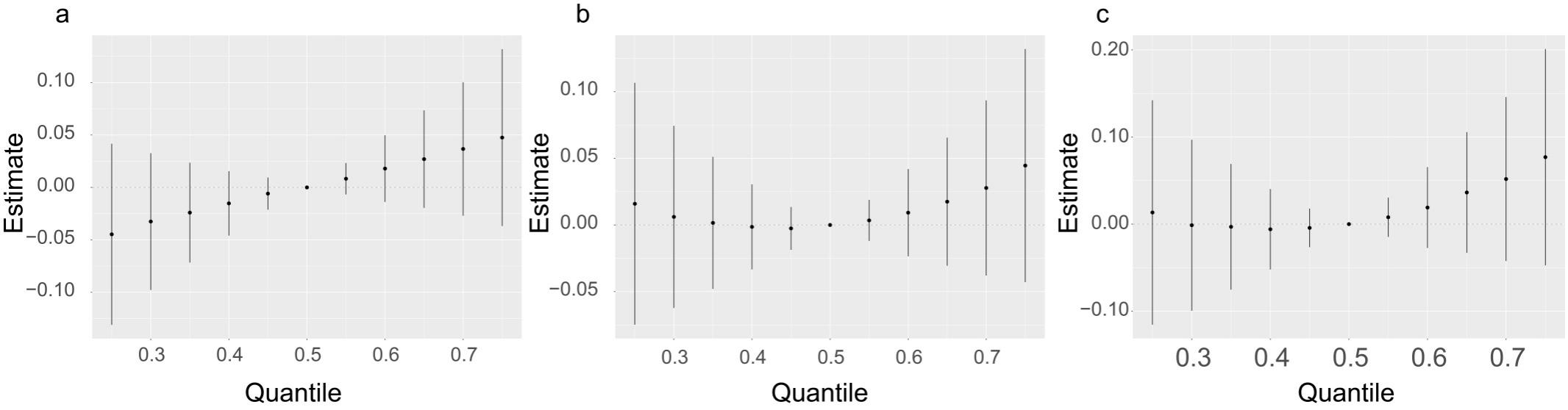

Supplement: Supplementary file 1 — (DOCX 306 kb) [file 11356_2023_27717_MOESM1_ESM.docx]
